# Proteomics Reveal Enhanced Oxidative Stress Responses and Metabolic Adaptation in *Acidithiobacillus ferrooxidans* Biofilm Cells on Pyrite

**DOI:** 10.3389/fmicb.2019.00592

**Published:** 2019-03-29

**Authors:** Sören Bellenberg, Dieu Huynh, Ansgar Poetsch, Wolfgang Sand, Mario Vera

**Affiliations:** ^1^Centre for Ecology and Evolution in Microbial Model Systems, Linnaeus University, Kalmar, Sweden; ^2^Biofilm Centre, Aquatische Biotechnologie, Universität Duisburg-Essen, Essen, Germany; ^3^Institute of Biosciences, TU Bergakademie Freiberg, Freiberg, Germany; ^4^Plant Biochemistry, Ruhr-University Bochum, Bochum, Germany; ^5^School of Biomedical and Healthcare Sciences, University of Plymouth, Plymouth, United Kingdom; ^6^College of Environmental Science and Engineering, Donghua University, Shanghai, China; ^7^Institute for Biological and Medical Engineering, Schools of Engineering, Medicine and Biological Sciences, Pontificia Universidad Católica de Chile, Santiago, Chile; ^8^Department of Hydraulic and Environmental Engineering, School of Engineering, Pontificia Universidad Católica de Chile, Santiago, Chile

**Keywords:** *Acidithiobacillus ferrooxidans*, proteomics, bioleaching, oxidative stress, reactive oxygen species, biofilm formation, pyrite

## Abstract

Reactive oxygen species (ROS) cause oxidative stress and growth inhibition by inactivation of essential enzymes, DNA and lipid damage in microbial cells. Acid mine drainage (AMD) ecosystems are characterized by low pH values, enhanced levels of metal ions and low species abundance. Furthermore, metal sulfides, such as pyrite and chalcopyrite, generate extracellular ROS upon exposure to acidic water. Consequently, oxidative stress management is especially important in acidophilic leaching microorganisms present in industrial biomining operations, especially when forming biofilms on metal sulfides. Several adaptive mechanisms have been described, but the molecular repertoire of responses upon exposure to pyrite and the presence of ROS are not thoroughly understood in acidophiles. In this study the impact of the addition of H_2_O_2_ on iron oxidation activity in *Acidithiobacillus ferrooxidans* DSM 14882^T^ was investigated. Iron(II)- or sulfur-grown cells showed a higher sensitivity toward H_2_O_2_ than pyrite-grown ones. In order to elucidate which molecular responses may be involved, we used shot-gun proteomics and compared proteomes of cells grown with iron(II)-ions against biofilm cells, grown for 5 days in presence of pyrite as sole energy source. In total 1157 proteins were identified. 213 and 207 ones were found to have increased levels in iron(II) ion-grown or pyrite-biofilm cells, respectively. Proteins associated with inorganic sulfur compound (ISC) oxidation were among the latter. In total, 80 proteins involved in ROS degradation, thiol redox regulation, macromolecule repair mechanisms, biosynthesis of antioxidants, as well as metal and oxygen homeostasis were found. 42 of these proteins had no significant changes in abundance, while 30 proteins had increased levels in pyrite-biofilm cells. New insights in ROS mitigation strategies, such as importance of globins for oxygen homeostasis and prevention of unspecific reactions of free oxygen that generate ROS are presented for *A. ferrooxidans* biofilm cells. Furthermore, proteomic analyses provide insights in adaptations of carbon fixation and oxidative phosphorylation pathways under these two growth conditions.

## Introduction

Biomining is the industrial application of acidophilic leaching microorganisms for the recovery of valuable metals from low-grade metal sulfide ores via bioleaching or biooxidation techniques. Biomining is currently used for the recovery of copper and gold from sulfide ores and accounts for 15–20% and 4–5% of the global copper and gold production, respectively (Brierley, 2016). These production processes, and also the recovery of other metals such as of zinc, nickel or cobalt via biomining techniques are established and expected to gain in importance for a sustainable resource supply (Brierley, 2016). Bioleaching technologies render low-grade ores, contaminated soils, industrial residues, and electronic waste materials, which are not used for established metal recovery processes to be valuable resources.

It is well accepted that biofilm formation of leaching microorganisms on metal sulfides is essential for bioleaching (Rohwerder et al., 2003; Vera et al., 2013b). The biofilm lifestyle is inherently connected with emergent properties (Flemming et al., 2016). As such, the accumulation of iron(III)-ions in within the extracellular polymeric substances (EPS) is of special importance for the mineral-oxidizing bacteria. Attached cells improve the leaching efficiency by forming a reaction space between the cells and the metal sulfide surfaces (Sand et al., 2001; Rohwerder and Sand, 2007). This reaction space is filled with EPS, which contain polysaccharides, lipids, proteins and uronic acid residues chelating iron-ions. Consequently, iron(III)-ions, which chemically attack the sulfide moiety of the metal sulfide, are abundant in the microbe-mineral interface. The re-generated iron(II)-ions are the electron donor for iron-oxidizing cells, that are the main drivers of bioleaching. Furthermore, cell–cell communication processes, and co-metabolism are emergent properties of biofilms that have been shown to occur within mixed species bioleaching cultures (Bellenberg et al., 2014; Smith and Johnson, 2018). It has been suggested these may significantly affect colonization of metal sulfides and leaching rates. Mixed species cultures can also increase the oxidative turnover of inorganic sulfur compounds (ISCs) and catalyze the reduction of iron(III)-ions ISC-oxidizing species of the acidithiobacilli (Marrero et al., 2015; Smith and Johnson, 2018). EPS production and biofilm formation in *Acidithiobacillus ferrooxidans* are processes known to be regulated by the energy source (Gehrke et al., 1998), the growth state [e.g., planktonic cells produce less EPS than biofilm ones (Gehrke et al., 1998; Bellenberg et al., 2012) and by quorum sensing (QS) (Gonzalez et al., 2012; Vera et al., 2013b; Bellenberg et al., 2014; Mamani et al., 2016)]. These factors extend also to biofilm formation of other *Acidithiobacillus*, *Leptospirillum* and *Acidiferrobacter* species. *N*-acyl homoserine lactones (AHL) specifically influence colonization of pyrite by different leaching bacteria, such as *A. ferrooxidans* (DSM 14882^T^), *Acidithiobacillus ferrivorans* SS3 (DSM 17398), *Acidithiobacillus ferriphilus* R1 (29444), *Acidithiobacillus thiooxidans* DSM 14887^T^, *Leptospirillum ferrooxidans* DSM 2931, *Acidiferrobacter thiooxydans* (DSM 2392^T^), and *Acidiferrobacter* sp. *SPIII/3* (DSM 27195) (Bellenberg et al., 2014). Current knowledge suggests intra- and interspecies cell-cell communication, mediated by AHLs and possibly also by other families of signaling compounds such as diffusible signal factor (DSF) family compounds, to exist in acid mine drainage (AMD) and bioleaching habitats (Valenzuela et al., 2007; Bellenberg et al., 2014, 2018). In addition, certain growth conditions such as enhanced ionic strength, lack of inorganic phosphate (P_i_), presence of sub-inhibitory amounts of chemicals such as chloride or copper ions, and growth temperature influence biofilm formation processes in iron-oxidizing acidithiobacilli (Bellenberg et al., 2015). It has also been shown that cell attachment and biofilm formation in bacteria is influenced by the second messenger c-di-GMP, which regulates several bacterial behaviors and is of key importance for driving the lifestyle switch between motile, planktonic cells and EPS-producing, biofilm cells (Hengge, 2009; Romling et al., 2013). Genes involved in c-di-GMP metabolism are abundant in the acidithiobacilli, and enhanced levels of c-di-GMP have been observed in biofilm forming cells of *A. ferrooxidans*^T^, *Acidithiobacillus caldus* (ATCC 51756^T^) and *A. thiooxidans* (DSM 14887^T^) (Ruiz et al., 2011; Castro et al., 2015; Díaz et al., 2018). In addition, regulation of swarming motility has also been correlated with lowered levels of c-di-GMP in *A. caldus*^T^ (Castro et al., 2015) and enhanced expression of the c-di-GMP effector protein PelD, which is involved in the regulation of EPS production, has been correlated with enhanced levels of c-di-GMP in *A. thiooxidans*^T^ (Díaz et al., 2018).

Acidophilic iron-oxidizing bacteria are confronted with high levels of reactive oxygen species (ROS) in their habitats. ROS include superoxide anions (O_2_^−^), hydroxyl radicals (OH^•^) and hydrogen peroxide (H_2_O_2_). ROS levels are enhanced in AMD ecosystems due to several reasons. High amounts of redox-active metals such as iron, copper, manganese and others are solubilized due to acid generation by microbial oxidation of inorganic sulfur compounds (ISC) to sulfuric acid. These metals are involved in Fenton and Haber–Weiss reactions generating ROS. In addition, ROS arise from surface reactions on metal sulfides, mainly due to reactions of molecular oxygen in acidic aqueous solutions with crystal lattice-bound iron or other metals (Cohn et al., 2006). H_2_O_2_ formation from pyrite slurries in the absence of dissolved oxygen has been also demonstrated (Borda et al., 2001). Grinding and crushing of metal sulfides bearing ores before biohydrometallurgical processing enlarges their surface areas, turning them to be accessible to foster dissolution reactions but also to be especially reactive regarding ROS generation. Physical processing, i.e., mechanical stress on crystal structures induces the formation of extremely reactive secondary transformation phases such as szomolnokite, a soluble iron sulfate secondary oxidation product (Eymery and Ylli, 2000). These oxidation/transformation processes are shown to affect sulfide minerals reactivity in acidic solutions, having a significant effect on ROS generation (Jones et al., 2013). Other metal sulfides than pyrite are also able to generate ROS. A decreasing order in their generation of H_2_O_2_ has been established for pyrite (FeS_2_), chalcopyrite (CuFeS_2_), sphalerite (ZnS) and galena (PbS), respectively (Nooshabadi and Rao, 2014). ROS such as O_2_^−^ and H_2_O_2_ are able to penetrate cell membranes in acidic environments at diffusion limited rates, causing macromolecule damage, when elevated concentrations of these compounds occur (Imlay, 2013). The inhibitory effects of ROS originating from sulfide mineral surfaces have been demonstrated with mesophilic and thermophilic leaching microorganisms (Jones et al., 2011; Ma and Lin, 2013).

In general, oxidative stress responses of bacteria have been observed to be part of complex phenomena such as biofilm formation, antibiotic resistance and pathogenicity (Cárdenas et al., 2012). In order to cope with high amounts of ROS, acidophilic iron-oxidizing bacteria possess a repertoire of adaptations (Ferrer et al., 2016b), including several constitutive ROS scavenging systems, which are considered to be the first defense barrier. In addition, a secondary defense barrier consists on the induced expression of specific ROS degrading enzymes and repair systems for damaged macromolecules (Cabiscol et al., 2000). Among the enzymes involved in oxidative stress responses, NADH-dependent oxidases have been suggested to reduce intracellular ROS generation (Cárdenas et al., 2012). Superoxide dismutase enzymes convert O_2_^−^ to H_2_O_2_ and O_2_ (Niederhoffer et al., 1990). Their encoding genes have been found to be present in most acidophilic leaching bacteria, with *Leptospirillum* spp. being an exception (Cárdenas et al., 2012). Catalases are enzymes widely known to be responsible for degradation of H_2_O_2_. These enzymes are not encoded in genomes of acidithiobacilli and leptospirilli. Consequently, alternative mechanisms for intracellular H_2_O_2_ degradation must be present in these species. It has been hypothesized that in acidophilic bacteria the AhpCF alkyl-hydroperoxidase/peroxiredoxin couple is not only involved in detoxification of organic peroxides but also in degradation of H_2_O_2_ (Ferrer et al., 2016b). Rubrerythrin has been found encoded in several *Leptospirillum* spp. genomes and suggested to function as H_2_O_2_ reductase (Maaty et al., 2009). Spermidine is a natural polyamine involved in crucial molecular processes such as DNA stability, transcription, translation, apoptosis, cell proliferation, differentiation and survival and has also been associated with protection against H_2_O_2_ (Minois et al., 2012). A protective effect against ROS has been attributed to spermidine and spermidine synthases, found in genomes of leptospirilli and acidithiobacilli (Valdes et al., 2008; Ferrer et al., 2016a). In addition, cobalamin (vitamin B_12_), a cobalt-containing tetrapyrrole cofactor, has recently been shown to be involved in attenuation of ROS generation in *Leptospirillum* sp. Group II CF-1 (Ferrer et al., 2016c).

Thiol groups of proteins and low molecular weight thiol compounds are also ROS scavengers. Consequently, protein repair systems such as the thioredoxin (TRX) and thiol/disulfide interchange systems are important for reestablishment of the intracellular redox balance. Glutathione (GSH), in addition to its role in sulfur oxidation (Rohwerder and Sand, 2003), is suggested to play together with GSH reductases, an important role in intracellular redox balance in *A. ferrooxidans*. A previous high-throughput proteome study showed that several proteins related to GSH metabolism had enhanced levels in early-stage biofilm formation of *A. ferrooxidans*^T^ on pyrite (Vera et al., 2013a).

The presence of pyrite has an inhibitory effect on the iron oxidation activity by iron(II) ion-grown *A. ferrooxidans* cells. This observation was previously suggested to be attributed to the enhanced presence of ROS in pyrite cultures (Bellenberg et al., 2015). In this study the levels of H_2_O_2_ generated in pyrite bioleaching assays in acidic medium were quantified in relation to the mineral grain size, the pulp density, and the presence of *A. ferrooxidans* cells. In addition, the concentration and exposure time-dependent influence of H_2_O_2_ on iron(II)-oxidation activity of *A. ferrooxidans* cells previously grown on iron(II) ions, elemental sulfur or pyrite was compared. In order to contribute to the understanding of molecular ROS defense mechanisms in this species and the adaptation of iron(II) ion-grown cells to grow with pyrite, proteomes of planktonic, iron(II) ion-grown cells and biofilm cells on pyrite grains, after 5 days of incubation were studied. Proteomic insights into the molecular mechanisms of oxidative stress management, biofilm formation, energy metabolism and carbon fixation are discussed.

## Materials and Methods

### Microbial Strains and Growth Conditions

*Acidithiobacillus ferrooxidans*^T^ DSM 14882 was grown in Mackintosh (MAC) basal salt medium (Mackintosh, 1978), either with iron(II) ions, elemental sulfur or pyrite as energy source. Iron(II)- or pyrite-grown cells were cultivated at pH 1.8 with 54 mM (3 g/L) iron(II) ions, supplied as FeSO_4_ × 7 H_2_O, or with pyrite grains (5% w/v, 50–100 or 100–200 μm grain size), respectively. Sulfur-grown cells were cultivated with elemental sulfur powder (1%, w/v) at an initial pH of 3.5.

### Pyrite Preparation

Pyrite grains were prepared from museum-grade pyrite cubes (Navajun mine, Spain). These were crushed with a disk swing-mill (HSM 100M, Herzog). After crushing, pyrite grains were wet sieved (Test sieves, Retsch, Germany). The particle size fraction between 50 and 100 μm was used in leaching experiments. For analysis of chemical H_2_O_2_ generation by pyrite grains, the fractions with 50–100 and 100–200 μm grain size were compared. Pyrite grains were boiled for 30 min in approximately 10 volumes of 6 M HCl, and then washed with deionized water until the pH was neutral. Afterwards grains were stirred twice in approximately five volumes of acetone for 30 min, in order to remove soluble sulfur compounds (Moses et al., 1987; Schippers et al., 1999). Acetone residues in pyrite samples were evaporated in a fume hood at room temperature for 12 h. The pyrite was sealed under a nitrogen atmosphere and sterilized for 24 h at 120°C.

### Photometric Quantification of H_2_O_2_ and Iron Ions

H_2_O_2_ was spectrophotometrically quantified as described (Baga et al., 1988). Concentrations of iron(II) ions and total iron were quantified using the 1,10-phenathrolin method (Harvey et al., 1955).

### Iron(II)-Oxidation Assays

Iron(II)-oxidation assays were prepared in 50 mL MAC medium (pH 1.8). Cells were harvested by centrifugation at 7,000 g at room temperature and washed with MAC medium (pH 1.8) before inoculation. Cultures were incubated at 28°C on a rotary shaker (120 rpm). In order to test the sensitivity of cells, regarding inhibition of iron(II)-oxidation activity upon exposure to H_2_O_2_, two types of assays were done. In the first one, iron(II)-, elemental sulfur- or pyrite-grown cells were treated for 24 h with varying H_2_O_2_ levels (0, 0.1, 0.5, and 1, 5 mM), at constant initial cell numbers (5 ⋅ 10^7^ cells/mL). In the second assay type, 5 ⋅ 10^7^ cells/mL of either iron(II)- or pyrite-grown cells were exposed to 1 mM H_2_O_2_ for different time periods (0, 2, 6, 12, and 24 h). In both cases, after preincubation with H_2_O_2_, iron(II) ions were added at 54 mM directly into the assay flasks and iron oxidation was measured. Experiments were done in triplicate.

### Pyrite Dissolution Assays

Pyrite-grown cells of *A. ferrooxidans* were inoculated in 50-mL cultures at 5% w/v, 50–100 μm pyrite grain size. Sterile controls were also prepared. Assays were incubated at 28°C with shaking at 120 rpm. Samples were taken periodically every 2–3 days for determination of iron(II) and iron(III) ion concentrations. The effect of externally added H_2_O_2_ (0.1, 0.5, and 1 mM) on pyrite dissolution of pyrite-grown cells of *A. ferrooxidans* was studied. The additions of H_2_O_2_ were 0.1 mM at days 0, 6, 12, and 18. On days 22 and 24 0.5 mM H_2_O_2_ was added and at day 26 the addition was 1 mM H_2_O_2_. The influence of external H_2_O_2_ addition on pyrite dissolution and cell growth was compared with control assays and without H_2_O_2_ addition. Experiments were done in triplicate.

### Cultivation Conditions, Protein Extraction and Proteomic Analysis

Proteomes from iron(II) ion-grown *A. ferrooxidans* cells were compared to those of cells after 5 days of biofilm formation on pyrite grains. A period of 5 days after the inoculation of pyrite cultures with iron(II) ion-grown cells is the time typically observed in our laboratory bioleaching experiments, until the planktonic cell number in pyrite cultures starts to increase. Iron(II) ion-grown cells were cultivated in 1-L Erlenmeyer flasks with 400 mL MAC medium (pH 1.8) with 54 mM iron(II)-ions. Cells were harvested at the late exponential growth phase and centrifuged at 7,000 *g*, washed with MAC medium and subjected to protein extraction using hot-phenol extraction as described below, according to (Vera et al., 2013a), or inoculated at a cell density of 5 ⋅ 10^7^ cells/mL to 5% pyrite cultures. These were prepared in 1-L Erlenmeyer flasks with 400 mL MAC medium (pH 1.8) and 20 g pyrite grains (50–100 μm). After 5 days of incubation at 120 rpm and 28°C, proteins of biofilm cells on pyrite grains were extracted. Briefly, cell pellets from iron(II)-grown cells or 20 g colonized pyrite grains were washed with 25 mL MAC medium and centrifuged for 5 min at 5,000 × *g*. Then, samples were incubated with 10 mL of cell lysis solution (20 mM sodium acetate pH 5.5, 1% SDS, 2 mM EDTA), for 10 min at 65°C with 15 s vortexing each min. After this step, 10 mL of acidic phenol (Sigma) were added and the mixture was incubated at 65°C for 10 min with 15 s vortexing each min. Proteins dissolved in the phenol phase were washed twice with one volume of H_2_O for 10 min at 70°C (with vortexing each 2 min). After each washing step, samples were chilled for 10 min on ice and centrifuged at 5,000 × *g* for 10 min at 4°C. Afterwards, the aqueous phase was discarded and proteins dissolved in the phenol phase were precipitated by addition of 1.5 volumes of ice-cold acetone. Samples were mixed and incubated at −20 °C overnight. Precipitated proteins were collected by centrifugation at 5,000 × *g* for 10 min at 4°C and washed twice with cold acetone. After evaporating the residual acetone, protein samples were dried for 20 min at room temperature and subjected to SDS-PAGE. Recovered peptides were subjected to LC-ESI-MS/MS analysis on a nanoAcquity UHPLC (Waters) coupled to an LTQ-XL Orbitrap (Thermo) system as described in detail in Vera et al. (2013a and references therein). Three independent samples were analyzed for each condition.

Proteins were identified with Andromeda and quantified with the label free quantification (LFQ) algorithm, embedded in MaxQuant version 1.5.5.1 (Cox et al., 2014). The following parameters were used: main search max. peptide mass error of 4.5 ppm, tryptic peptides of at least five amino acid length with maximally two missed cleavages, LFQ min. ratio count of two, matching between runs enabled, peptide spectrum matches (PSM) and (Razor) protein false discovery rate (FDR) of 0.01, advanced ratio estimation and second peptides enabled.

Spectra were searched against *A. ferrooxidans* database NC_011761.1 from Genbank. In addition, spectra were searched against a database of common exogenous protein contaminants provided by MaxQuant. Statistical analysis of proteome data was done with Perseus version 1.5.5.3 (Tyanova et al., 2016).

### Nucleic Acid Staining and Confocal Laser Scanning Microscopy

Cells used for comparative proteomic analyses were visualized. Iron(II)-grown cells were mounted on polycarbonate filters (GTTB, Ø 2.5 cm, 0.22 μm, Millipore^®^) washed with MAC medium and sterile-filtered tap water. Subsequently, cells were stained using a 6 μM solution of Syto^®^ 9 (Invitrogen^©^) for 15 min. In a comparable manner, biofilm cells on pyrite grains were washed and stained in reaction tubes containing 50 μL pyrite grains. Filters or pyrite grains were mounted with cover glasses on glass slides using Citifluor^®^ AF2. A laser scanning module (LSM 510 Carl Zeiss^®^ Jena) coupled to an inverted Axiovert100MBP microscope (Zeiss^®^) was used. For specific detection of Syto^®^ 9 fluorescence an argon laser at 488 nm and a 505–550 nm bandpass filter was used at constant excitation laser energy and detector settings. Micrographs were obtained with a plan-neofluar 100×/1.3 oil objective. The microscope was operated with the software LSM 510 Release 3.2 (Zeiss^®^).

**Table 1:** Effect of grain size and pulp density on H_2_O_2_ generation after 24 h incubation without inoculation in MAC medium.

Grain size (μm)	Pulp density % (w/v)		

	5	10	30
		H_2_O_2_ [mM]
**50–100**	0.17 ± 0.01	0.29 ± 0.01	0.83 ± 0.06
**100–200**	0.05 ± 0.00	0.09 ± 0.01	0.25 ± 0.01

## Results

### Development of H_2_O_2_ Concentration in Pyrite-Containing Media

**FIGURE 1:**
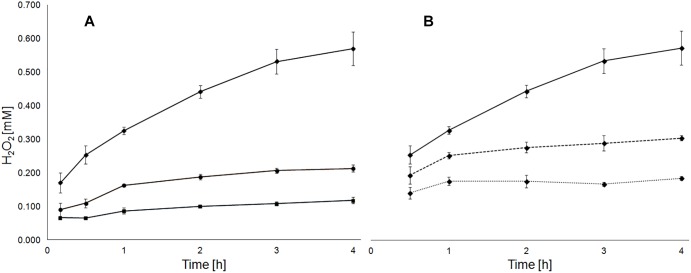
Pulp density and presence of *A. ferrooxidans* cells influence the generation of H_2_O_2_ in medium with pyrite. Development of H_2_O_2_ concentration in medium with 5 (boxes), 10 (circles) and 30% pyrite (rhombs) with a grain size of 50–100 μm **(A)**. Development of H_2_O_2_ concentration in medium with 30% pyrite without (solid line) and with presence of *A. ferrooxidans* cells at 5 ⋅ 10^7^ (dashed line) or 10^8^ (dotted line) cells/mL **(B)**.

The concentration of H_2_O_2_ after 24 h exposure of pyrite grains with different particle sizes was quantified (Table 1). Pulp density positively correlated with the amount of generated H_2_O_2_. Consequently, pyrite with 50–100 μm grain size generated higher concentrations of H_2_O_2_ than the preparation with 100–200 μm grain size. Likewise, at a pulp density of 5%, 0.17 ± 0.01 mM H_2_O_2_ were generated within 24 h in MAC medium with pyrite of 50–100 μm grain size while nearly five times higher levels of H_2_O_2_ were observed in assays with pulp density of 30% (w/v). The H_2_O_2_ scavenging effect of bacterial cells at a pyrite pulp density of 30% is shown in Figure 1B.

**Table 2:** H_2_O_2_ concentration-dependent inhibition of iron oxidation by cells of *A. ferrooxidans* after 24 h preincubation^∗^ with H_2_O_2_.

Pre-cultivation substrate	Hydrogen peroxide [mM]				
	0	0.1	0.5	1	5
	Time [days] after treatment with H_2_O_2_, when biological iron oxidation was evident (Supplementary Figure S1)				
Iron(II) ions	0	9	9	–	–
Elemental sulfur	1	2	6	8	–
Pyrite	2	2	6	8	–

*^∗^24 h preincubation with H_2_O_2_ without addition of electron donors. (−) No biological iron oxidation was observed after 2 months. The data were derived from spectrophotometric measurements of iron(II)-ions and total iron ions. Details are presented in Supplementary Figure S1.*

### Differential Sensitivity of Iron(II)-, Elemental Sulfur- and Pyrite-Grown Cells to the Presence of H_2_O_2_

Iron(II) ion-, elemental sulfur- or pyrite-grown cells were compared regarding their ability to oxidize iron(II) ions after 24 h exposure to H_2_O_2_, without external addition of electron donors (Table 2). After H_2_O_2_ treatment, 54 mM iron(II)-ions were added to the assays. Chemical iron(II) ion oxidation by H_2_O_2_ via the Fenton reaction occurred immediately after its addition, accounting for 4% (2.1 mM ± 0.1 mM) and 16% (8.8 ± 0.8 mM) of the iron(II) ions, to the assays that were initially supplied with 1 and 5 mM H_2_O_2_, respectively.

In assays with pyrite- or sulfur-grown cells, biological iron oxidation occurred after exposure to 1 mM H_2_O_2_. Iron(II) ion-grown cells were more sensitive than pyrite- or sulfur-grown cells and did not exhibit iron-oxidation activity after 24 h exposure to H_2_O_2_ concentrations higher than 0.5 mM. In general, a strong decrement in biological iron(II)-oxidation rates was evident in assays where cells were pre-incubated with 0.1 and 0.5 mM H_2_O_2_. In these assays, iron oxidation occurred after a lag-phase of 6–9 days, while control experiments without addition of H_2_O_2_ exhibited biological iron oxidation after a lag-phase of 1 or 2 days (Table 2).

**Table 3:** Time-dependent inhibition of iron oxidation by cells of *A. ferrooxidans* after preincubation^∗^ with 1 mM H_2_O_2_.

Pre-cultivation substrate	Exposure time [h]				
	0	2	6	12	24
	Time [days] after treatment with H_2_O_2_, until biological iron oxidation was observed (Supplementary Figure S2)				
Iron(II) ions	1	2	4	–	–
Pyrite	2	5	7	7	7

*^∗^Pre-incubation was done without addition of electron donors. (−) no biological iron oxidation was observed after 2 months. The data were derived from spectrophotometric measurements of iron(II)-ions and total iron ions. Details are presented in Supplementary Figure S2.*

In agreement with the dose dependent effects of H_2_O_2_, a clear trend of enhanced inhibition of iron(II) oxidation activity with prolonged exposure times was also observed (Table 3). Pyrite-grown cells showed an increased tolerance to prolonged exposure to 1 mM H_2_O_2_ compared to iron(II) ion-grown cells.

### Adaptation to ROS Is Inherent if *A. ferrooxidans* Grows on Pyrite as Energy Source

**FIGURE 2:**
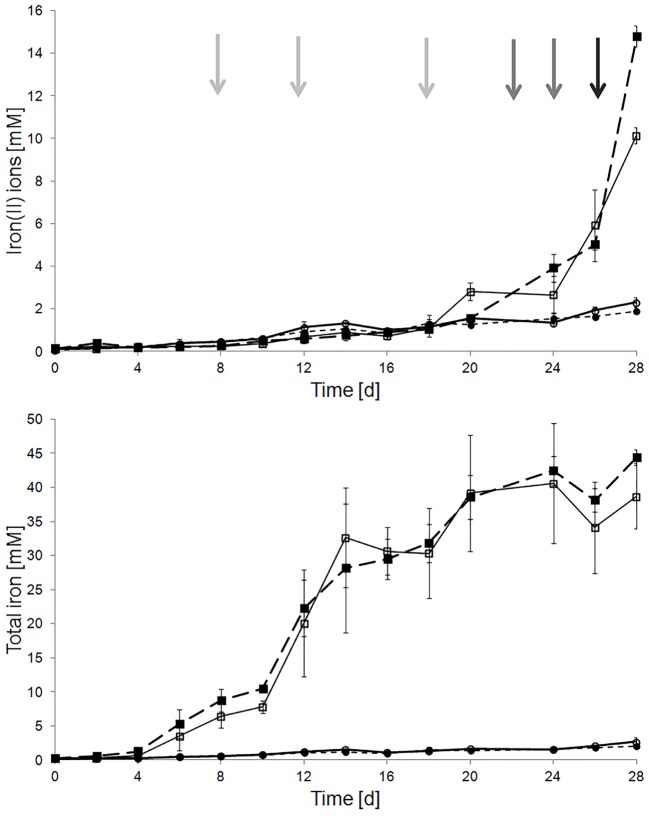
Effect of external addition of H_2_O_2_ on pyrite dissolution by pyrite-grown cells of *A. ferrooxidans*. Total and iron(II) ion concentrations were measured in sterile (*circles*) and inoculated (*squares*) pyrite dissolution assays, with (*empty symbols*) and without (*filled symbols*) periodic H_2_O_2_ addition. Added H_2_O_2_ concentrations were 0.1 mM at days 0, 6, 12, and 18 (*gray arrows*), 0.5 mM at days 22 and 24 (*dark gray arrows*), and 1 mM at day 26 (*black arrow*). Assays were incubated at 28 °C with agitation (120 rpm). The inoculum size was 5 ⋅ 10^7^ cells/mL.

Pyrite dissolution assays with pyrite-grown cells of *A. ferrooxidans* and sterile controls were characterized by total iron concentrations of 44.41 ± 1.07 and 1.96 ± 1.43 mM after 28 days, respectively. In addition, there were no differences in pyrite dissolution in assays with or without repetitive addition of 0.1, 0.5, or 1 mM of H_2_O_2_ regarding the development of the iron(II) ion and total iron concentration (Figure 2). These results indicate the presence of efficient molecular mechanisms mitigating the inhibitory effects of extracellular hydrogen peroxide in cells grown with pyrite as sole electron donor.

### Proteomic Analysis of Adaptation of Iron(II) Ion-Grown Cells to Growth With Pyrite

**FIGURE 3:**
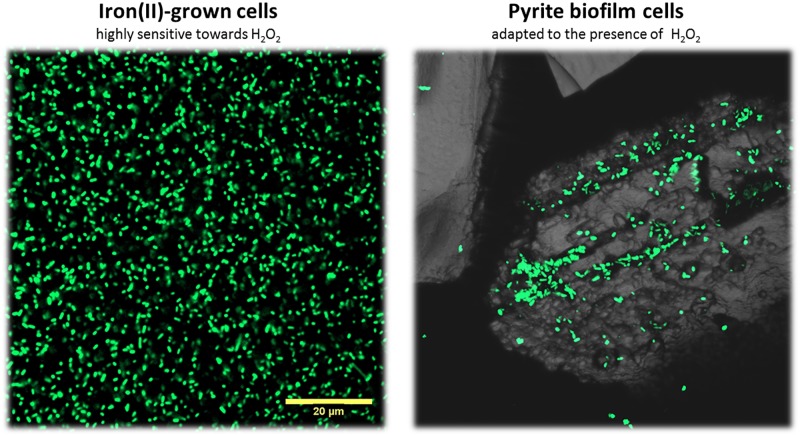
Confocal laser scanning microscopy of iron(II)-grown cells and pyrite-biofilm cells of *A. ferrooxidans*^T^. Iron(II)-grown cells from liquid cultures were filtered on a polycarbonate membrane prior staining with the nucleic acid dye Syto 9. Biofilm cells were stained directly on the pyrite grains. Both cells types were compared in our proteome analyses. Maximum intensity projections are shown. The size bar measures 20 μm and is applicable to both images.

In order to identify the molecular nature of oxidative stress responses upon biofilm formation of *A. ferrooxidans* on pyrite, among other responses, we compared proteomes from 5 days old biofilm cells on pyrite against cells grown on ferrous iron as energy source (Figure 3). In total, 1157 proteins were found (Supplementary Proteome Dataset). According to a functional classification in clusters of orthologous groups (COGs), these were distributed in 21 functional categories (Figure 4.1). After statistical analysis for strong changes on protein abundance levels (log2 > |1|, *q*-value ≤ 0.05), 420 proteins were characterized as differentially expressed between the two growth conditions. From this group, 213 (Figure 4.3) and 207 (Figure 4.4) proteins had increased levels in iron(II) ion-grown cells and in 5 day-old pyrite biofilm cells, respectively. The shared proteome, consisting of proteins that are not differentially expressed among both cell populations (Figure 4.2) consists of 737 proteins and is characterized by a very similar percentile representation of COGs, as in the complete proteome. In general, the results indicated that iron(II) ion-grown cells showed enhanced levels of proteins related to growth metabolism, such as ribosomal proteins, oxidative phosphorylation, carbon fixation, nucleotide metabolism and sulfate assimilation. Proteins assigned to the COG “translation” (J), containing ribosomal proteins, represented the largest COG of proteins with increased levels (20%) in iron(II) ion-grown cells. In comparison, proteins in this COG represented only 2% of the ones with increased levels in biofilm cells. In contrast, pyrite biofilm cells showed increased amounts of proteins involved in formate, pyruvate, carbohydrate, and lipopolysaccharide metabolism. Proteins clustered in the COG “carbohydrate transport and metabolism” (G) had enhanced levels in pyrite biofilm cells (7%), and were less represented among proteins with increased levels in iron(II) ion-grown cells (3%). A similar observation was made with proteins in the COG secondary metabolites biosynthesis, transport, and catabolism (Q) with 4 and 1% of enhanced proteins in pyrite biofilm and iron(II) ion-grown cells, respectively.

**FIGURE 4:**
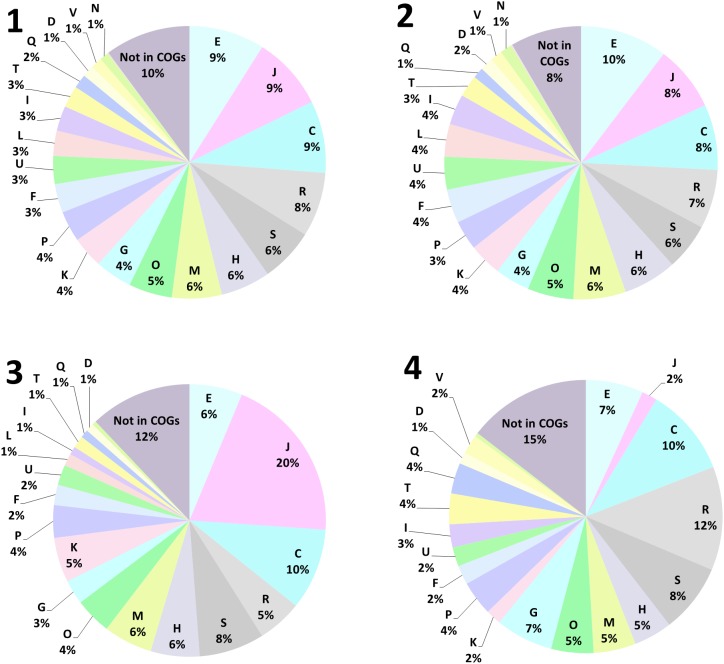
Functional annotation of proteins found in iron(II)-grown and 5-day-old biofilm cells of *A. ferrooxidans*, using clusters of orthologous groups (COG). In total 1157 proteins were found **(1)**, 737 proteins were shared among both populations in the core proteome **(2)**. 213 proteins were found increased in late exponential growth-phase iron(II)-grown cells **(3)**, while 207 proteins were found increased in 5-day-old biofilm cells on pyrite grain **(4)**. Cell cycle control, cell division, chromosome partitioning (D), Cell wall/membrane/envelope biogenesis (M), Cell motility (N), Post-translational modification, protein turnover, and chaperones (O), Signal transduction mechanisms (T), Intracellular trafficking, secretion, and vesicular transport (U), Defense mechanisms (V), Extracellular structures (W), Nuclear structure (Y), Cytoskeleton (Z), RNA processing and modification (A), Chromatin structure and dynamics (B), Translation, ribosomal structure and biogenesis (J), Transcription (K), Replication, recombination and repair (L), Energy production and conversion (C), Amino acid transport and metabolism (E), Nucleotide transport and metabolism (F), Carbohydrate transport and metabolism (G), Coenzyme transport and metabolism (H), Lipid transport and metabolism (I), Inorganic ion transport and metabolism (P), Secondary metabolites biosynthesis, transport, and catabolism (Q), General function prediction only (R), Function unknown (S).

Proteins that were either not assigned to a COG or belong to poorly characterized ones, such as “function unknown” (S) or “general function prediction only” (R), represent a large fraction of the proteins with increased levels in biofilm cells (35%), and in iron(II) ion-grown cells (25%). These were also present within the shared core proteome (21%). Consequently, still a large fraction of unknown proteins probably accounts for phenotypic differences between iron(II)- and pyrite-grown biofilm cells.

Proteins assigned to the COG “energy production and conversion” (C) accounted for 10% of the whole pool of increased proteins in both cell types. The main differences observed in this category were in proteins related to ISCs oxidation, which were found increased in biofilm cells (Supplementary Table S1).

### Proteins Related to Iron and Sulfur Metabolism

The fact that iron(II) ion-grown cells are more actively growing than biofilm cells after 5 days of contact to pyrite, without a soluble electron donor, is supported by enhanced levels of proteins associated with assimilatory sulfate reduction (Supplementary Table S1), such as sulfate adenylyltransferase (Sat, AFE_0539; CysD, AFE_3124; CysN, AFE_3125), phosphoadenosine phosphosulfate reductase (CysH, AFE_3123) and sulfite reductase (CysJ, AFE_3121; CysI, AFE_3122).

Proteins encoded in gene clusters involved in iron(II) ion oxidation in *A. ferrooxidans* were found in both growth conditions (Supplementary Table S1). During growth on iron(II) ions several proteins assembling the *bc*1 complex, including ubiquinol-cytochrome *c* reductase (PetA-1, AFE_3109), ubiquinol cytochrome *c* oxidoreductase (PetC-1, AFE_3111) and cytochrome *b* (PetB-1, AFE_3110), were found to be strongly enhanced. Furthermore, rusticyanin (AFE_3146), cytochromes *c*552, *b*561 and *c*4 (AFE_3152, AFE_2328, AFE_3107), and proteins involved in cytochrome biogenesis (AFE_3112, AFE_3113) were also found enhanced. The cytochrome *c* oxidase subunits I and II (CoxA and CoxB, AFE_3149, _3150) were also found enhanced, while cytochrome *c* (AFE_3153) and subunits of the NADH:ubiquinone oxidoreductase (AFE_2621 – AFE 2629) were found at similar levels in both growth conditions.

In contrast, proteins associated with ISC oxidation had enhanced levels in pyrite biofilm cells (Supplementary Table S1). These include tetrathionate hydrolase (AFE_0029), the DoxD family quinol oxidase (AFE_0044) and proteins encoded in its genomic context (AFE_0043 and AFE_0045). Also the cytochrome *o* ubiquinol oxidase (AFE_0631 – AFE_0633), cytochrome *d*/*bd* ubiquinol oxidase subunits I and II (AFE_0954 and AFE_0955), sulfide/quinone reductase (AFE_1792), sulfur reduction protein DsrE (AFE_2548), heterodisulfide reductase subunits (AFE_2551, AFE_2554, AFE_2555, and AFE_2586), and several pyridine nucleotide-disulfide oxidoreductases (AFE_0859, AFE_1803, and AFE_2553), were found to be increased in these cells. Enhanced levels of enzymes involved in GSH metabolism, including GSH synthase (GshB, AFE_3063) and GSH sulfur transferase (AFE_2594), besides being involved in protection against oxidative stress, are probably contributing to the increased turnover of ISC in pyrite biofilm cells. This confirms observations suggesting that biofilm cells are preferentially oxidizing ISC, rather than iron (II) ions (Vera et al., 2009). It is known that *A. ferrooxidans*^T^ cells repress the expression of ISC oxidation pathways in presence of iron(II) ions as shown by comparing gene expression patterns of planktonic, iron(II) ion-grown cells against sulfur-grown ones using DNA-microarrays (Quatrini et al., 2006; Quatrini et al., 2009).

### Proteins Related to General and Oxidative Stress Management

In total, 80 proteins potentially involved in stress responses were found. These proteins are involved in general stress responses such as chaperones, heat- or cold-shock and metal resistance, as well as proteins involved in specific ROS defense mechanisms such as ROS degradation, macromolecule repair or stabilization, thiol compound redox balance as well as control of intracellular levels of metals and oxygen (Supplementary Table S2). Based on the statistical analysis of differential expression (|1| > log_2_, *q*-value ≤ 0.05), 42 proteins were found to be present in cells under both cultivation conditions, suggesting a “housekeeping oxidative stress management” role for these proteins. In contrast, 30 proteins (Supplementary Table S2) were found to be enhanced in biofilm cells. A globin (AFE_0861) was the most increased protein in pyrite biofilm cells. This suggests that the control of free molecular oxygen levels by oxygen-binding globins may be of importance for prevention of intracellular ROS formation by side-reactions of flavoenzymes during aerobic respiration and via Haber–Weiss reactions of redox active metals in biofilm cells. Several proteins involved in heavy metal transport, e.g., a copper-translocating P-type ATPase (AFE_2021), a heavy metal-binding protein (AFE_1862), ferritins (AFE_1682 and AFE_2347) as well as the arsenate reductase ArsC (AFE_2860), presumably involved in export of arsenite and antimonite, were found to be increased in biofilm cells. Several proteins involved in redox balance and protein repair mechanisms were also found to be enhanced in biofilm cells. Among those were a thioredoxin (AFE_2867), two thiol reductase thioredoxins (AFE_1979 and AFE_2590), two pyridine nucleotide-disulfide oxidoreductases (AFE_0859 and AFE _1803), three peroxiredoxins (AFE_0363, AFE_3241, and AFE_3116), two ABC exporter subunits CydC (AFE_1390, AFE_1388) and a thiol:disulfide interchange protein (AFE_0545). Also, the iron donor protein CyaY (AFE_2195), involved in biosynthesis and repair of FeS-cluster proteins (Ai et al., 2011), was found to be strongly increased in biofilm cells.

In addition, several universal stress (AFE_0751, AFE_2183, and AFE _2259), and heat- and cold-shock proteins (AFE_2086, AFE_0871, AFE_0590, and AFE_1648), were found to be increased in pyrite biofilm cells. AFE_1648 and AFE_2086 are chaperones from the Hsp20 superfamily. Both have been also found to be induced in *A. ferrooxidans* upon cultivation with U(VI) (Dekker et al., 2016), while AFE_2086 has been found induced after Cu^2+^ addition to planktonic iron (II) grown cells (Almárcegui et al., 2014). Furthermore, proteins involved in DNA repair mechanisms such as DNA exonuclease PolX (AFE_3104), restriction endonuclease EcoEI (AFE_0684), the DNA repair protein RecN (AFE_0450), were found to be increased in biofilm cells.

In iron(II) ion-grown cells only eight proteins associated with functions related to protection against oxidative stress or macromolecule repair were found to be enhanced. As such, the exodeoxyribonuclease X (AFE_1767) and the molecular chaperone DnaJ (AFE_2664) were found increased in iron(II) ion-grown cells. Other increased proteins involved in redox balance in iron(II) ion-grown cells include a glutathione amide-dependent peroxidase (AFE_0367) and two disulfide isomerases (AFE_1943 and AFE_2246). Cobalamin has been described as an antioxidant in *Leptospirillum ferriphilum* (Ferrer et al., 2016c). However, the enhanced abundance of the cobalamin biosynthesis protein CbiX (AFE_3127) found in this study in iron(II) ion-grown cells of *A. ferrooxidans* may rather be primarily connected with growth metabolism, which was observed to be enhanced in comparison to the pyrite biofilm cells population, by analysis of the proteome data. Furthermore, other globin (AFE_1548) and ferritin (AFE_0199) proteins were found to be more abundant in iron(II) ion-grown, planktonic cells.

Taken together, the presented data provide clear evidence that the biofilm cell subpopulation is expressing a strong additional repertoire of active molecular responses to cope with enhanced levels of ROS. These include ROS degradation, redox balance, DNA and protein repair mechanisms, as well as metal and oxygen homeostasis. Altogether, these responses may contribute to the adaptation to the presence of elevated levels of ROS during growth on pyrite. This positively correlates with an enhanced tolerance of pyrite-grown cells to stress by addition of H_2_O_2,_ as shown in Tables 2, 3.

### Transcriptional Regulators

Twenty-two transcriptional regulators and transcription factors were identified based on previous reports in *A. ferrooxidans* (Valdes et al., 2008; Hödar et al., 2012; Vera et al., 2013a). Of those proteins, nine were found enhanced in biofilm cells, and fourteen in iron(II) ion-grown cells (Supplementary Table S3). Biofilm cells had enhanced levels of three nitrogen fixation related regulatory proteins (AFE_0024, AFE_0429, and AFE_2915). The gene encoding for AFE_2915 has been found induced when *A. ferrooxidans* cells were treated with the QS analog tetrazole 9c, and suggested to be involved in the uptake of nitrogen when forming biofilms (Mamani et al., 2016). Furthermore, in those cells a transcriptional regulator (AFE_2557), located in genomic context of sulfur metabolism related genes (AFE_2547–AFE_2558) was found to be enhanced. This protein seems to be unique in *Acidithiobacillus.* A LysR family transcriptional regulator (AFE_0135), a cold-shock protein, also annotated as an ATP dependent RNA helicase (AFE_0590), were also enhanced in biofilm cells. In iron(II) ion-grown cells transcription factors, such as the transcription initiation protein Tat (AFE_1689), the translation initiation factor IF-2 (AFE_0391) and the sigma factor RpoD (AFE_2336), were among the enhanced ones. Interestingly, the IclR family transcriptional regulator (AFE_1668) was found enhanced in iron(II) ion-grown cells. The same protein was found to be enhanced in biofilm cells, compared to its planktonic cell subpopulation in pyrite cultures, during *A. ferrooxidans* early biofilm formation stage (24 h) (Vera et al., 2013a).

### Proteins Related to Carbon and Carbohydrate Metabolism and Biofilm Formation

Several proteins related to CO_2_ fixation were identified in both growth conditions (Supplementary Table S4). The Cbb1 RuBisCo subunits and most of the proteins, which are encoded in the putative *cbb1* operon structure (AFE_1676–AFE_1691), were enhanced in iron(II) ion-grown cells. The exception represents carboxysome and carbon dioxide concentrating proteins (AFE_1683–AFE_1687), which were found enhanced in pyrite biofilm cells. In addition, Cbb2 RuBisCo proteins, encoded in the putative *cbb2* operon (AFE_3051 – AFE_3054), were also found to be enhanced. The remaining Calvin cycle enzymes encoded in the *cbb* operon, shown in Supplementary Table S4, had no significant changes in their abundance levels, with the exception of ribulose-5-phosphate isomerase (AFE_0629) and a transketolase (AFE_1843) that were strongly enhanced in pyrite biofilm cells.

Proteins associated with pyruvate metabolism were also more abundant in pyrite biofilm cells. As well, enhanced cystein and methionine biosynthesis may be inferred from increased levels of methylene-tetrahydrofolate reductase (AFE_0535) and *S*-adenosylmethionine synthase in these cells, while enhanced carbon fixation may correlate with elevated levels of phosphoribulokinase (AFE_0536) observed in iron(II) ion-grown cells.

In Supplementary Table S5 the fraction of differentially expressed proteins in pyrite biofilm and iron(II) ion-grown cells in COGs “cell wall/membrane biogenesis” (M), “carbohydrate transport and metabolism” (G), “intracellular trafficking and secretion” (U), “lipid transport and metabolism” (I) and cell motility (N), which represent 26% and 20% of these 170 proteins, respectively, are shown. Among those were fifteen enhanced proteins in biofilm cells belonging to the group “carbohydrate transport and metabolism” (G), compared to six proteins in iron(II) ion-grown cells. Proteins of this category, which were enhanced in iron(II) ion-grown cells, are involved in carbon fixation and central carbon metabolism, such as RuBisCo (AFE_1691), transketolase (AFE_1667), aldolases (AFE_1667 and AFE_1766), pyruvate kinase (AFE_3249) and phosphoglucosamine mutase (AFE_2634). Interestingly, proteins increased in iron(II) ion-grown cells of the category “intracellular trafficking and secretion” (U) are proteins, such as the flagellar motor protein MotA (AFE_2270), proteins involved in protein secretion (AFE_1398, AFE_0247, AFE_2856, and AFE_3049) and pilus assembly (AFE_0736 and AFE_0737). However, also in biofilm cells, proteins with similar functional associations were found increased within this COG. Namely, the pilus assembly protein PilZ (AFE_1915), known to represent a c-di-GMP effector domain containing protein, was enhanced in biofilm cells, as well as three RND proteins (AFE_1880; AFE_3264; and AFE_2367). RND proteins comprise seven families of bacterial efflux pumps, that are normally involved in cell homeostasis, removal of toxic compounds such as heavy metals and toxins (Anes et al., 2015).

Some proteins found increased in biofilm cells, are directly related to carbohydrate biosynthesis and modification, such as a sucrose synthase (AFE_1552), catalyzing sucrose biosynthesis from UDP-glucose and D-fructose, a PTS sugar transporter (AFE_3023), a PTS sugar transporter subunit IIA (AFE_3020), both probably involved in phosphorelay chains for phosphorilation of sugars, concomitant to their translocation across the cell membrane. The release of sugars may be occurring through an alpha-glucan phosphorylase (AFE_1799), probably involved in the cleavage of an alpha 1,4 linkage from polymers such as maltodextrin or glycogen, as well as through a glycogen branching enzyme (AFE_2836), which could provide branched chains to glycogen phosphorylases. Three different glucose dehydrogenases were found, a putative glucose 1-dehydrogenase (AFE_0748), a glucose dehydrogenase (AFE_1857), a glucose-6-phosphate dehydrogenase (AFE_2025). The first two enzymes are probably involved in generating D-gluconate, while the third one generates D-gluconate-6-phosphate. These can be precursors for pyruvate, ribulose-5 phosphate or glyceraldehyde. A group 1 glycosyltransferase (AFE_2967), probably involved in the biosynthesis of EPS precursors was also found enhanced in biofilm cells.

## Discussion

The mineral surface area-dependent generation of H_2_O_2_ was described (Jones et al., 2013), and its occurrence in laboratory pyrite dissolution assays and consequently also its relevance in industrial bioleaching applications is known. In this article the levels of H_2_O_2_ were quantified, in order to demonstrate its relevance for the proteomic responses of biofilm cells in pyrite bioleaching experiments done in this study, and in order to define experimental conditions for H_2_O_2_ cell exposure experiments. *A. ferrooxidans* biomass clearly scavenges H_2_O_2_. The sensitivity of living cells to H_2_O_2_ was demonstrated to be dependent on the pre-cultivation substrate, and the adaptation of cells to pyrite. In case of pyrite-grown cells, external addition of 100 μM H_2_O_2_ had no effect on pyrite dissolution. However, the addition of 1 mM H_2_O_2_ at the inoculation stage caused a significant reduction in pyrite colonization by pyrite-grown cells of *A. ferrooxidans* (Bellenberg et al., 2015). This observation can be explained by the detrimental effect of 1 mM H_2_O_2_ that we observed with iron(II)- and pyrite-grown cells. However, cells adapted to grow using pyrite as energy source were less sensitive than iron(II) ion-grown cells to externally added H_2_O_2_ or to pyrite-generated ROS. This observation is in agreement with increased amounts of proteins associated with an oxidative stress response, intracellular redox balance and macromolecule repair mechanisms, found in proteomes of biofilm cells. Several *A. ferrooxidans* enzymes catalyzing ROS degradation are expressed apparently in a constitutive manner between iron (II) and pyrite grown cells, and were therefore not enhanced significantly between these growth conditions. In contrast, thiol/disulfide redox balance systems such as thioredoxins and their respective reductases which are suggested to be especially important for the oxidative stress management in *L. ferriphilum* (Norambuena et al., 2012), were overrepresented in the enhanced proteins in pyrite biofilm cells of *A. ferrooxidans*. Iron-sulfur clusters are prone to oxidative damage. In this context the FeS cluster assembly protein IscX (AFE_0679) as well as the iron donor protein CyaY (AFE_2195) (Ai et al., 2011), were found to be strongly enhanced in biofilm cells. This observation suggests an increased synthesis and probably also repair of FeS-cluster proteins to be important in biofilm cells. The ROS defense strategy of *A. ferrooxidans* is amended by expression of metal-binding proteins and O_2_-binding globin-like proteins, which are suggested to control the intracellular levels of free metal ions and oxygen. Protective functions of globins are due to O_2_-binding, and hence, prevention of intracellular generation of ROS due to oxygen reactions with reduced flavoenzymes or Haber–Weiss reactions with free redox active metal ions (Herold and Fago, 2005), contributing to prevention of oxidative damage in *A. ferrooxidans.* Our results indicate that the importance of globins for oxygen supply may be extended to a function of protection from undesired oxidative reactions. A globin (AFE_0861) was the most increased protein with a log_2_ expression ratio of 6.9 in the pyrite biofilm cell population. Another globin (AFE_1548) was found enhanced in iron(II) ion-grown cells (log_2_ = 1.5). The globin, which was found enhanced in biofilm cells appears to have high similarities with proteins annotated as flavohemoproteins. Consequently, it can be suggested that this protein may be, besides its role in O_2_-binding and transport, functional as a nitric oxide dioxygenase, protecting cells from oxidative and nitrosative stress and mediating NO-signaling (Gardner et al., 1998). Likewise, the globin encoded in the gene AFE_1548, which was found enhanced in iron(II) ion-grown cells, is annotated as a truncated hemoglobin group 2 and, therefore also suggested to be directly involved in mitigation of oxidative and nitrosative stress. Several enzymes involved in the pentose phosphate pathway were found induced in biofilm cells, such as aldolase and transaldolase and several glucose dehydrogenases. The aldolases comprise a link between the glycolytic and the pentose phosphate pathways. Therefore, it is reasonable to assume that biofilm cells are increasing the levels of NADH and NADPH, generated in these pathways to cope with oxidative stress. Furthermore, our proteomic comparison highlights the importance of other antioxidants, such as spermine/spermidine and GSH in iron(II) ion-grown and pyrite biofilm cells (AFE_0156, AFE_0366, AFE_0367, AFE_3038, and AFE_3063). The increased levels of GSH synthase (AFE_3063) in biofilm cells suggest an increase in periplasmic GSH levels, probably related to a GSH-driven response against oxidative stress, which has been reported to occur in biofilm cells upon contact to pyrite (Vera et al., 2013a). The GSH reductase encoding gene in *A. ferrooxidans* is also induced after cell exposure to copper, suggesting its involvement in recovering GSH pools, copper homeostasis and protection from copper induced oxidative stress (Xia et al., 2011; Almárcegui et al., 2014). In this study, metal exporters, including the copper translocating P-type ATPase (AFE_2021) and disulfide isomerases were found enhanced in pyrite biofilm cells. Likewise, decreased levels of the major outer membrane protein (Omp40) and some ionic transporters suggests that a decrease in the influx of metal cations might occur upon exposure to copper and, probably also to pyrite (Almárcegui et al., 2014).

In addition to the demonstrated adaptation to ROS upon cell exposure to pyrite, the presence of ISCs from pyrite clearly induced expression of genes related to ISC oxidation. The up-regulation of GSH synthase may also participate in sulfur metabolism, since activation of elemental sulfur by GSH is suggested to mediate its oxidation by the enzyme sulfur dioxygenase (Rohwerder and Sand, 2003). In iron(II) ion-grown *A. ferrooxidans* cells, enhanced levels of proteins associated with oxidative phosphorylation, iron(II) oxidation, reverse electron flow for generation of reducing power and carbon fixation, as well as protein biosynthesis suggest a global alignment of metabolism toward active growth, compared to cell maintenance metabolism, oxidative stress responses, membrane transport functions and macromolecule repair mechanisms in biofilm cells at the observed stage of development. The cytochrome *bc*1 complex was strongly enhanced in iron(II) ion-grown cells, which confirms the utilization of reverse electron flow during autotrophic growth with iron(II) ions. In comparison, pyrite biofilm cells, utilize ISCs, and therefore use the downhill electron flow for NAD(P)H biosynthesis (Nitschke and Bonnefoy, 2016). Enhanced amounts of most of the proteins encoded in the putative *cbb1* operon were observed in iron(II) ion-grown cells. However, other proteins encoded in this putative operon structure such as carboxysome peptides A and B (CsoS4A and CsoS4B) were even found enhanced in pyrite biofilm cells. Interestingly, the RuBisCo proteins CbbL2 and CbbS2 and the RuBisCo activation proteins CbbQ2 and CbbO2, encoded in the *cbb2* operon were increased in biofilm cells. This finding confirms a suggested differential expression of the different putative *cbb* operon structures under varying environmental conditions (Appia-Ayme et al., 1999; Esparza et al., 2010). These two RuBisCo enzymes are of type I, which is proposed to serve at current atmosphere conditions, namely low CO_2_ and high O_2_ concentrations, in combination with CO_2_-concentrating carboxysomes. In contrast, the RuBisCo type 2, encoded in the cbb5 operon (Appia-Ayme et al., 2006), is predicted to be used when increased concentrations of CO_2_ and reduced levels of O_2_ are available (Shively et al., 1998). This RuBisCo enzyme was also found to have similar levels in both cell populations. Taken together, the redundancy in RuBisCo genes and the different levels of proteins in both cell populations of the two different RuBisCo type 1 emphasizes a versatile adaptation to ensure CO_2_ fixation even under CO_2_ limited conditions. The different abundancies of RuBisCO proteins in iron(II) ion- and pyrite-exposed cells may also be correlated with the availability of ISCs. Elevated expression of both RuBisCo type 1 genes was measured for sulfur-grown cells compared to iron(II) ion-grown ones (Appia-Ayme et al., 2006; Esparza et al., 2010). This finding is reasonable considering that ISCs represent more energetic substrates than iron(II) ions. However, the proteomes presented here show enhanced amounts of phosphoribulokinase CbbP in iron(II) ion-grown cells, suggesting an enhanced carbon fixation activity in these cells, which show active growth compared to the proteome of pyrite biofilm cells. Cytochrome *bd* oxidase subunit I and cytochrome *d* ubiquinol oxidase subunit II assemble an enzyme that is relevant for O_2_ reduction at low levels of O_2_, demonstrated to be insensitive to sulfide inhibition (Forte et al., 2016). Its enhanced amount in pyrite biofilm cells may be related to environmental conditions of lowered O_2_ pressure in biofilm cells and also to the presence of toxic sulfide in pyrite cultures. Furthermore, cytochrome *bd* oxidase is known to reduce O_2_ to H_2_O efficiently without formation of ROS under low O_2_ partial pressure and, thereby, contributing to protection from oxidative stress, as demonstrated for the strictly anaerobic bacterium *Moorella thermoacetica* (Das et al., 2005). Efficient oxygen reduction without formation of ROS is also relevant for iron(II) ion-grown cells, which exhibited enhanced levels of the cytochrome *c* oxidase subunits I and II (CoxA and CoxB, AFE_3149, AFE_3150). This can therefore be considered a general mechanism in oxidative stress mitigation.

Interestingly, the transcriptional regulator Fur (AFE_0282), involved in iron homeostasis and consequently indirectly also oxidative stress response (Quatrini et al., 2005, 2007), was not found to be differentially expressed. Consequently, regulation of iron uptake is hypothesized to be equally relevant under both growth conditions in which iron is abundant. Therefore, the proposed role of Fur in oxidative stress responses (Quatrini et al., 2007) seems to be equally valid under both growth conditions and consequently other mechanisms must be involved in sensing and controlling enhanced levels of ROS from pyrite and other metal sulfide minerals.

## Conclusion

We have demonstrated that H_2_O_2_ occurs in pyrite bioleaching assays in concentrations that are inhibitory especially for iron(II)-grown cells of *A. ferrooxidans*. Pyrite-biofilm cells are less effected regarding their ability to oxidize iron(II)-ions after exposure to H_2_O_2_. The proteomic analysis of those different cells confirms enhanced levels of proteins with functions related to oxidative stress and detoxification of ROS. We highlight the importance of globins in combination with metal homeostasis mechanisms, proteins related with ROS degradation functions, repair mechanisms and production of EPS as parts of a multiple barrier and defense system against oxidative stress in biofilm cells of *A. ferrooxidans* growing on pyrite.

## Author Contributions

SB designed and supervised the experiments, wrote the manuscript, and analyzed the proteomic data. DH performed most of the experimental work. AP did proteomic measurements and processing of raw data. WS contributed to the redaction of the manuscript. MV contributed to the design of experiments, literature analysis, and writing the manuscript.

## Conflict of Interest Statement

The authors declare that the research was conducted in the absence of any commercial or financial relationships that could be construed as a potential conflict of interest.
