# Supplementary material for: Proteomics Reveal Enhanced Oxidative Stress Responses and Metabolic Adaptation in Acidithiobacillus ferrooxidans Biofilm Cells on Pyrite
Source: Front Microbiol. 2019 Mar 29;10:592. doi: 10.3389/fmicb.2019.00592 (PMC6450195; doi:10.3389/fmicb.2019.00592)
Supplement: Supplementary file 1 [file Data_Sheet_1.pdf]

Proteomics reveal enhanced oxidative stress responses and metabolic adaptation in *Acidithiobacillus ferrooxidans* biofilm cells on pyrite

Sören Bellenberg[1,2*], Dieu Huynh[3], Ansgar Poetsch[4,5], Wolfgang Sand [2,3,6], Mario Vera[7,8*]

***Correspondence:** soeren.bellenberg@lnu.se, mariovera@uc.cl

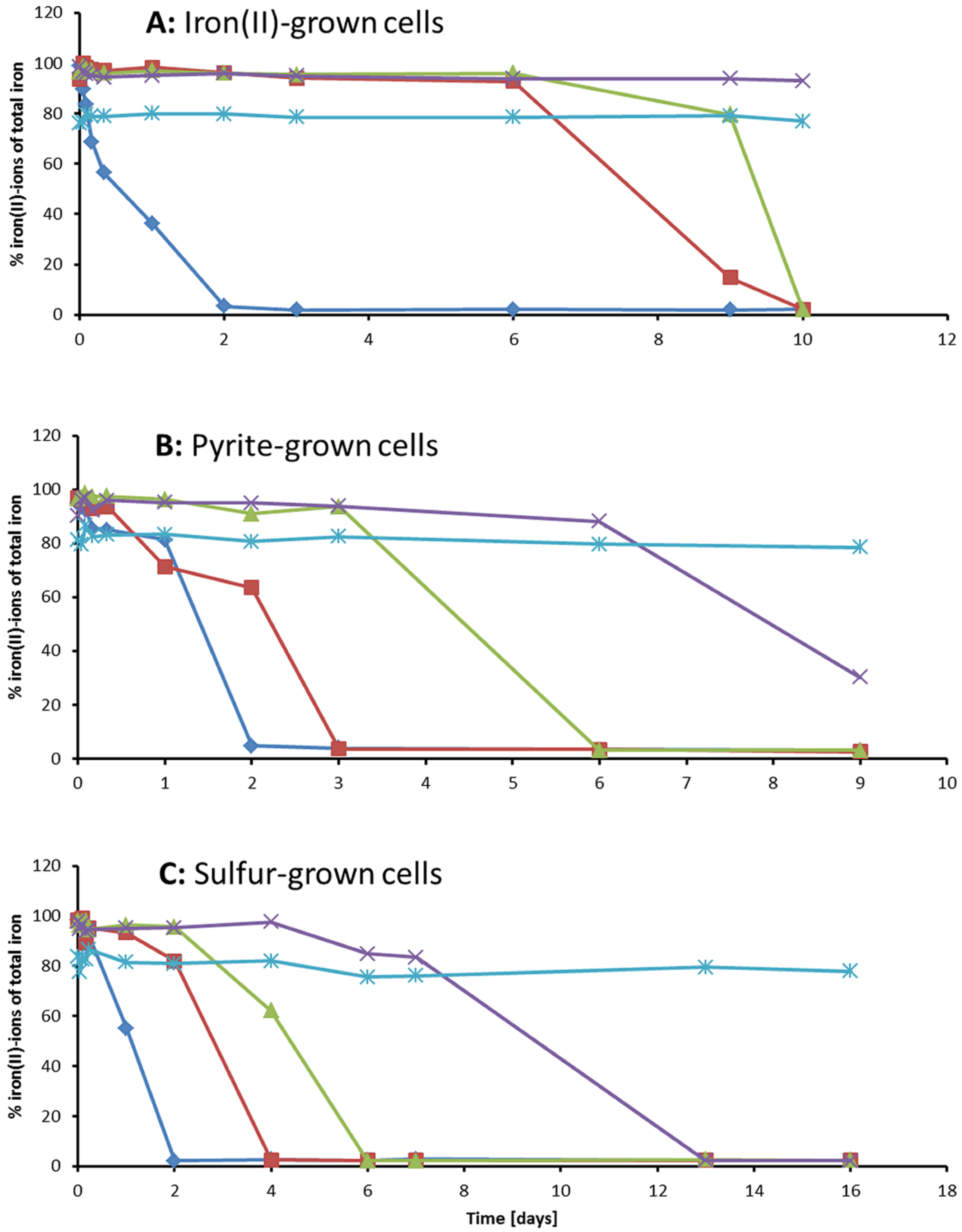

Supplemental Figure 1. The growth substrate influences *A. ferrooxidans*^{T} cells sensitivity towards H_2O_2. Before addition of the substrate iron(II)-ions (54 mM), iron(II)- (A), pyrite- (B) or sulfur-grown cells (C) were preincubated for 24 h with different concentrations of H_2O_2. The development of the percentile fraction of iron(II)-ions in assays with 0 (blue diamonds), 0.1 (red boxes), 0.5 (green triangles), 1 (violet crosses) and 5 mM H_2O_2 (light blue crosses) during the preincubation is shown.

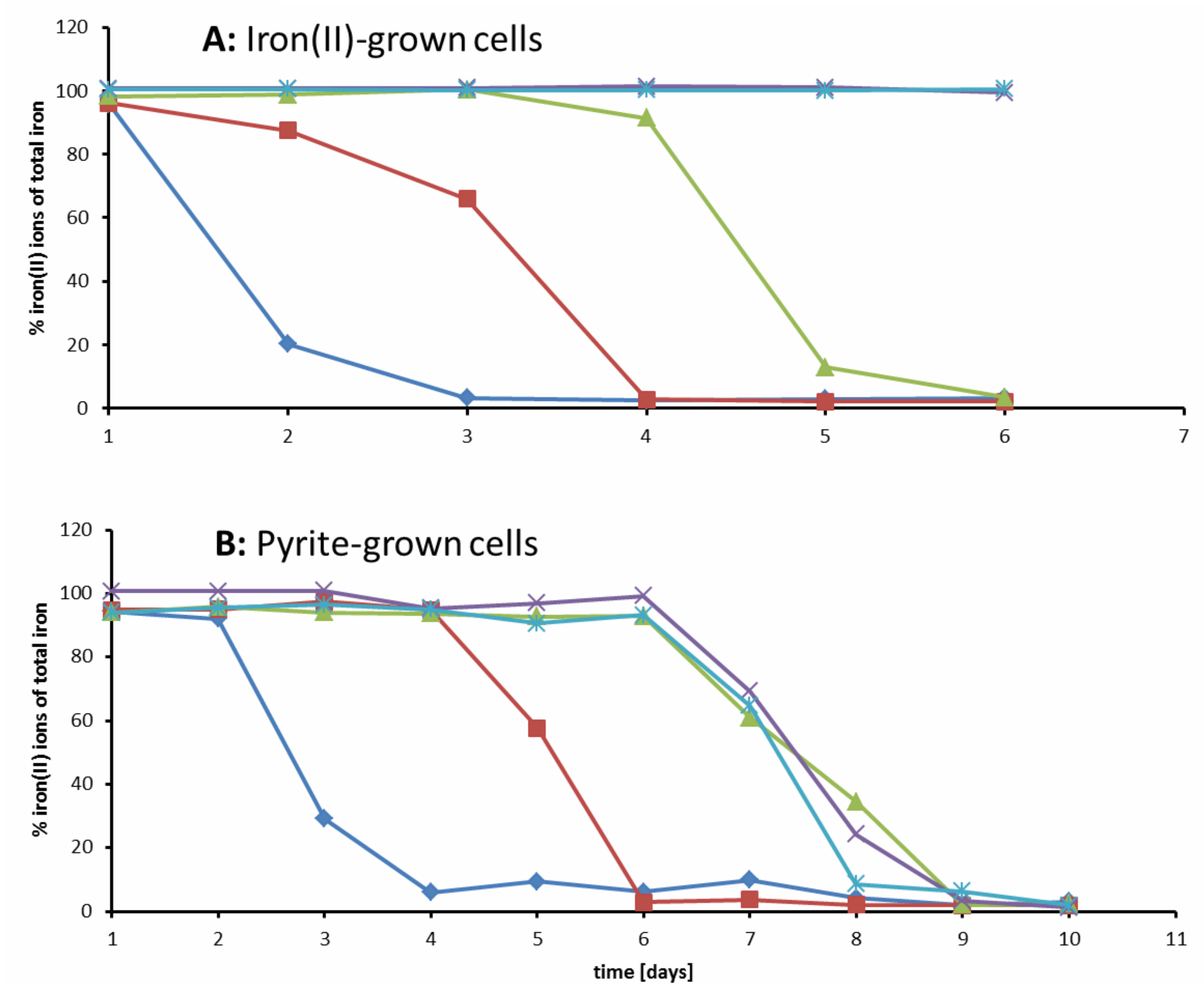

Supplemental Figure 2. Pyrite-grown cells are less sensitive to H_2O_2 than iron(II)-grown cells. The duration of exposure influences *A. ferrooxidans*^{T} cells sensitivity towards H_2O_2. Before addition of the substrate iron(II)-ions (54 mM), iron(II)- (A) or pyrite-grown cells (B) were preincubated for different periods of time with 1 mM H_2O_2. The development of the percentile fraction of iron(II)-ions in assays with 0 (blue diamonds), 2 (red boxes), 6 (green triangles), 12 (violet crosses) and 24 h (light blue crosses) preincubation is shown.
