# Supplementary material for: Proteomics Reveal Enhanced Oxidative Stress Responses and Metabolic Adaptation in Acidithiobacillus ferrooxidans Biofilm Cells on Pyrite
Source: Front Microbiol. 2019 Mar 29;10:592. doi: 10.3389/fmicb.2019.00592 (PMC6450195; doi:10.3389/fmicb.2019.00592)
Supplement: Supplementary file 2 [file Data_Sheet_2.pdf]

Proteomics reveal enhanced oxidative stress responses and metabolic adaptation in *Acidithiobacillus ferrooxidans* biofilm cells on pyrite

Sören Bellenberg[1,2*], Dieu Huynh[3], Ansgar Poetsch[4,5], Wolfgang Sand [2,3,6], Mario Vera[7,8*]

***Correspondence:** soeren.bellenberg@lnu.se, mariovera@uc.cl

Supplementary Table 1. Proteins of COG energy production and conservation (C) plus selected proteins associated with sulfur metabolism, iron(II)- and RISC oxidation in *A. ferrooxidans*^{T}

Locus tag	Protein ID	Description	log_2 pyrite/Fe	q-value
**AFE_0029**	WP_012535754.1	tetrathionate hydrolase	1.12	0.01
**AFE_0043**	WP_009566763.1	glycine/betaine ABC transporter substrate-binding protein	1.66	0.02
**AFE_0044**	WP_009566762.1	DoxD family quinol oxidase	2.55	0.00
**AFE_0045**	WP_009566760.1	sulfur/pyrite/thiosulfate/sulfide-induced protein	1.00	0.00
**AFE_0151**	WP_012535979.1	sulfurtransferase	3.98	0.00
**AFE_0177**	WP_012536002.1	glutathione S-transferase	0.69	0.03
**AFE_0267**	WP_009565299.1	sulfide/quinone oxidoreductase, sqr	0.44	0.19
**AFE_0366**	WP_012536110.1	glutathione-disulfide reductase	-0.03	0.98
**AFE_0423**	WP_012536129.1	aconitate hydratase	0.77	0.01
**AFE_0424**	WP_012536130.1	isocitrate dehydrogenase (NADP(+))	-0.28	0.15
**AFE_0425**	WP_009564695.1	succinyl-CoA ligase subunit beta	-0.24	0.26
**AFE_0427**	WP_012536131.1	succinyl-CoA synthetase subunit alpha	0.48	0.13
**AFE_0492**	WP_009566885.1	inorganic pyrophosphatase	-0.16	0.84
**AFE_0536**	WP_012536189.1	phosphoribulokinase	-0.97	0.03
**AFE_0539**	WP_012536192.1	adenylyl-sulfate kinase	-1,79	0.01
**AFE_0545**	WP_012536195.1	thiol:disulfide interchange protein	2.21	0.00
**AFE_0631**	WP_012536247.1	cytochrome o ubiquinol oxidase	5.03	0.00
**AFE_0632**	WP_012536248.1	cytochrome o ubiquinol oxidase	2.56	0.01
**AFE_0633**	WP_012536249.1	cytochrome o ubiquinol oxidase	0.73	0.48
**AFE_0660**	WP_009560818.1	malate dehydrogenase	1.44	0.09
**AFE_0674**	WP_009566457.1	iron-sulfur cluster scaffold-like protein	-1.96	0.01
**AFE_0678**	WP_009566453.1	ferredoxin, 2Fe-2S type, ISC system	-0.05	0.93
**AFE_0692**	WP_012536289.1	oxidoreductase alpha (molybdopterin) subunit	-0.35	0.29

**AFE_0697**	WP_012536293.1	S-(hydroxymethyl)glutathione dehydrogenase	-1.59	0.01
**AFE_0701**	WP_012536297.1	hydrogenase	-0.88	0.05
**AFE_0702**	WP_012536298.1	cytochrome-c3 hydrogenase	-1.01	0.00
**AFE_0808**	WP_009561607.1	oxidoreductase	0.99	0.01
**AFE_0809**	WP_012536374.1	oxidoreductase	-0.60	0.31
**AFE_0810**	WP_012536375.1	oxidoreductase	0.00	1.00
**AFE_0859**	WP_009564795.1	pyridine nucleotide-disulfide oxidoreductase	5.34	0.00
**AFE_0861**	WP_009564313.1	globin	6.87	0.00
**AFE_0954**	WP_012536460.1	cytochrome d ubiquinol oxidase subunit II	4.80	0.01
**AFE_0955**	WP_009567364.1	cytochrome bd oxidase subunit I	2.18	0.00
**AFE_0958**	WP_009565875.1	pyridine nucleotide-disulfide oxidoreductase	-0.76	0.03
**AFE_0984**	WP_012536482.1	ferredoxin	-0.50	0.17
**AFE_1388**	WP_012536491.1	thiol reductant ABC exporter subunit CydD	1.86	0.00
**AFE_1390**	WP_009566343.1	thiol reductant ABC exporter subunit CydC	1.92	0.00
**AFE_1546**	WP_012536595.1	NAD-dependent succinate-semialdehyde dehydrogenase	1.98	0.01
**AFE_1652**	WP_012607156.1	oxidoreductase molybdopterin subunit	0.58	0.06
**AFE_1662**	WP_009566373.1	NADH dehydrogenase	-1.89	0.00
**AFE_1663**	WP_009566374.1	glycolate oxidase iron-sulfur subunit	-1.63	0.00
**AFE_1664**	WP_012607162.1	glycolate oxidase subunit GlcE	-0.07	0.96
**AFE_1683**	WP_012607172.1	carbon dioxide-concentrating protein CcmK	0.14	0.83
**AFE_1685**	WP_012536700.1	carbon dioxide-concentrating protein CcmK	-0.25	0.31
**AFE_1686**	WP_009567525.1	carboxysome peptide B	0.97	0.08
**AFE_1687**	WP_009567526.1	carboxysome peptide A	1.03	0.71
**AFE_1690**	WP_009567528.1	ribulose bisphosphate carboxylase small subunit	-1.44	0.01
**AFE_1723**	WP_009563486.1	general stress protein	0.30	0.07
**AFE_1769**	WP_012536743.1	FAD-linked oxidase	-1.40	0.03
**AFE_1792**	WP_012536761.1	sulfide:quinone reductase	1.01	0.00
**AFE_1803**	WP_009568931.1	pyridine nucleotide-disulfide oxidoreductase	3.10	0.01
**AFE_1808**	WP_012536772.1	phosphate butyryltransferase	2.28	0.05
**AFE_1810**	WP_012607242.1	acetate kinase	-0.49	0.58
**AFE_1811**	WP_012536774.1	2-oxo acid dehydrogenase acyltransferase	1.26	0.01

**AFE_1812**	WP_012536775.1	pyruvate dehydrogenase subunit beta	0.97	0.00
**AFE_1813**	WP_009563431.1	dehydrogenase	0.78	0.07
**AFE_1844**	WP_009561766.1	ferredoxin	1.83	0.01
**AFE_1883**	WP_012536819.1	phosphoenolpyruvate carboxylase	-2.14	0.00
**AFE_1893**	WP_012536825.1	dihydrolipoyl dehydrogenase	2.81	0.00
**AFE_2075**	WP_012536951.1	3-isopropylmalate dehydrogenase	0.83	0.00
**AFE_2120**	WP_012536987.1	hopanoid biosynthesis associated radical SAM protein HpnJ	-1.51	0.00
**AFE_2126**	WP_012536992.1	glycerol-3-phosphate dehydrogenase (NAD(P)(+))	-0.60	0.22
**AFE_2131**	WP_012536996.1	aldehyde dehydrogenase	0.62	0.05
**AFE_2211**	WP_012537054.1	oxidoreductase	-0.33	0.64
**AFE_2256**	WP_012537084.1	MBL fold metallo-hydrolase	-0.13	0.70
**AFE_2321**	WP_012537120.1	monooxygenase	0.24	0.69
**AFE_2328**	WP_012537125.1	cytochrome b561	-1.86	0.00
**AFE_2364**	WP_009562958.1	sulfurtransferase	-2.31	0.10
**AFE_2388**	WP_012537164.1	2-octaprenyl-6-methoxyphenyl hydroxylase	0.42	0.22
**AFE_2389**	WP_012537165.1	2-octaprenyl-6-methoxyphenyl hydroxylase	-1.29	0.01
**AFE_2481**	WP_012607441.1	mercury(II) reductase	1.03	0.00
**AFE_2533**	WP_012537244.1	oxidoreductase	0.34	0.11
**AFE_2547**	WP_012537252.1	glycine cleavage system protein H	-0.01	0.98
**AFE_2548**	WP_012537253.1	sulfur reduction protein DsrE	3.45	0.01
**AFE_2549**	WP_009565261.1	glycine cleavage system protein H	0.58	0.13
**AFE_2550**	WP_012537254.1	disulfide reductase	1.27	0.00
**AFE_2551**	WP_012607468.1	heterodisulfide reductase subunit C	0.57	0.13
**AFE_2552**	WP_012537255.1	hypothetical protein	0.76	0.08
**AFE_2553**	WP_012537256.1	pyridine nucleotide-disulfide oxidoreductase	1.81	0.00
**AFE_2554**	WP_049756725.1	heterodisulfide reductase subunit B	0.83	0.04
**AFE_2555**	WP_041647471.1	heterodisulfide reductase subunit C	2.27	0.00
**AFE_2556**	WP_009562304.1	NADH dehydrogenase	1.61	0.00
**AFE_2557**	WP_009562307.1	transcriptional regulator	3.06	0.00
**AFE_2558**	WP_012537258.1	sulfurtransferase	-0.84	0.10
**AFE_2586**	WP_012537276.1	heterodisulfide reductase subunit B	1.53	0.00
**AFE_2594**	WP_012537282.1	glutathione S-transferase	0.86	0.01
**AFE_2621**	WP_009565427.1	NADH:ubiquinone oxidoreductase subunit J	1.07	0.17
**AFE_2622**	WP_012537299.1	NADH-quinone oxidoreductase	0.01	0.99

		subunit I		
**AFE_2623**	WP_009565424.1	NADH-quinone oxidoreductase subunit H	0.06	0.94
**AFE_2624**	WP_012537300.1	NADH-quinone oxidoreductase subunit G	0.20	0.23
**AFE_2625**	WP_012537301.1	NADH-quinone oxidoreductase subunit F	-0.43	0.11
**AFE_2626**	WP_009566653.1	NADH dehydrogenase subunit E	-0.27	0.80
**AFE_2627**	WP_012537302.1	NADH-quinone oxidoreductase subunit D	-0.22	0.14
**AFE_2628**	WP_009566652.1	NADH-quinone oxidoreductase subunit C	-0.06	0.91
**AFE_2629**	WP_009566651.1	NADH-quinone oxidoreductase subunit B 2	-0.96	0.06
**AFE_2673**	WP_009565770.1	class II fumarate hydratase	0.03	0.89
**AFE_2959**	WP_009564008.1	Fe(2+)-trafficking protein	-0.74	0.23
**AFE_3052**	WP_009566929.1	ribulose bisphosphate carboxylase small subunit	2.55	0.00
**AFE_3065**	WP_012537576.1	citrate synthase	0.27	0.46
**AFE_3068**	WP_012537578.1	pyruvate dehydrogenase	0.92	0.00
**AFE_3069**	WP_012537579.1	pyruvate dehydrogenase subunit beta	-0.34	0.27
**AFE_3070**	WP_012537580.1	pyruvate dehydrogenase (acetyl-transferring) E1 component subunit alpha	-0.69	0.03
**AFE_3079**	WP_009567865.1	nitroreductase	1.70	0.02
**AFE_3107**	#NV	cytochrome c4, CycA-1	-3.49	0.00
**AFE_3108**	WP_009566406.1	short-chain dehydrogenase, Sdr_1	-2.51	0.00
**AFE_3109**	#NV	ubiquinol--cytochrome c reductase, iron-sulfursubunit, PetA-1	-9.49	0.00
**AFE_3110**	WP_009566409.1	cytochrome b, PetB-1	-4.85	0.00
**AFE_3111**	WP_012537599.1	ubiquinol cytochrome C oxidoreductase, PetC-1	-6.93	0.00
**AFE_3112**	WP_012537600.1	cytochrome c biogenesis protein, ResB	-1.48	0.00
**AFE_3113**	WP_009566490.1	c-type cytochrome biogenesis protein CcsB, ResC	-2.33	0.00
**AFE_3121**	WP_012537605.1	sulfite reductase subunit alpha	-0.98	0.15
**AFE_3122**	WP_009566801.1	sulfite reductase subunit beta	-9.44	0.00
**AFE_3123**	WP_012537606.1	phosphoadenosine phosphosulfate reductase	-5.48	0.00
**AFE_3124**	WP_041646660.1	sulfate adenylyltransferase small subunit	-6.87	0.00
**AFE_3125**	WP_012537608.1	sulfate adenylyltransferase	-7.98	0.00
**AFE_3146**	WP_012537622.1	rusticyanin	-1.79	0.00
**AFE_3149**	WP_009561084.1	cytochrome c oxidase subunit I,	-0.72	0.07

		coxA		
**AFE_3150**	WP_009561085.1	cytochrome c oxidase subunit II, coxB	-0.86	0.01
**AFE_3151**	WP_012537626.1	hypothetical protein	-0.63	0.01
**AFE_3152**	WP_012537627.1	cytochrome c552	-1.58	0.00
**AFE_3153**	WP_012537628.1	cytochrome c	-0.17	0.64
**AFE_3161**	WP_009562388.1	lactate dehydrogenase	1.52	0.00
**AFE_3202**	WP_009569104.1	F0F1 ATP synthase subunit epsilon	-0.87	0.28
**AFE_3203**	WP_012537655.1	ATP synthase subunit beta	-0.21	0.54
**AFE_3204**	WP_012537656.1	ATP synthase subunit gamma	-3.75	0.00
**AFE_3205**	WP_012537657.1	ATP synthase subunit alpha	0.66	0.03
**AFE_3206**	WP_009561112.1	ATP synthase subunit delta	-0.35	0.58
**AFE_3207**	WP_009561113.1	ATP synthase subunit B	0.16	0.63
**AFE_3238**	WP_009567147.1	ferredoxin	-1.32	0.45
**AFE_3286**	WP_012537694.1	hydrogenase 2 large subunit	2.54	0.02

Supplementary Table 2. Proteins with functions related to oxidative stress management and repair mechanisms

Locus tag	Protein ID	Description	log_2 pyrite/Fe	q-value
**AFE_0861**	WP_009564313.1	globin	6.87	0.00
**AFE_0859**	WP_009564795.1	pyridine nucleotide-disulfide oxidoreductase	5.34	0.00
**AFE_3104**	WP_012537597.1	DNA polymerase/3'-5' exonuclease PolX	4.58	0.00
**AFE_0751**	WP_009564668.1	universal stress protein	4.23	0.02
**AFE_1862**	WP_009568883.1	heavy metal-binding protein	3.53	0.03
**AFE_2867**	WP_012537441.1	thioredoxin	3.51	0.00
**AFE_2183**	WP_012537036.1	universal stress protein	3.36	0.00
**AFE_2195**	WP_009567357.1	iron donor protein CyaY	3.29	0.01
**AFE_2021**	WP_012536912.1	copper-translocating P-type ATPase	3.15	0.00
**AFE_1803**	WP_009568931.1	pyridine nucleotide-disulfide oxidoreductase	3.10	0.01
**AFE_2259**	WP_009562362.1	universal stress protein	2.76	0.01
**AFE_0363**	WP_012536107.1	peroxiredoxin	2.68	0.00
**AFE_2086**	WP_009567699.1	heat-shock protein Hsp20	2.29	0.00
**AFE_0871**	WP_012536407.1	heat-shock protein Hsp20	2.24	0.19
**AFE_1970**	WP_012536875.1	ATP-dependent chaperone ClpB	2.22	0.00
**AFE_0545**	WP_012536195.1	thiol:disulfide interchange protein	2.21	0.00
**AFE_0590**	WP_009564993.1	cold-shock protein	2.13	0.05
**AFE_1979**	WP_012536882.1	thiol reductase thioredoxin	2.04	0.00
**AFE_1390**	WP_009566343.1	thiol reductant ABC exporter subunit CydC	1.92	0.00
**AFE_1648**	WP_009569199.1	heat-shock protein Hsp20	1.86	0.00
**AFE_1388**	WP_012536491.1	thiol reductant ABC exporter subunit CydD	1.86	0.00
**AFE_3241**	WP_009567144.1	peroxiredoxin	1.86	0.02
**AFE_0450**	WP_012536142.1	DNA repair protein RecN	1.79	0.00
**AFE_2375**	WP_012537158.1	ATP-dependent helicase	1.74	0.01
**AFE_2590**	WP_012537279.1	thiol reductase thioredoxin	1.72	0.13
**AFE_0178**	WP_012536003.1	exodeoxyribonuclease III	1.64	0.35

**AFE_2945**	WP_012537491.1	peptide-methionine (R)-S-oxide reductase	1.44	0.02
**AFE_1408**	WP_012536503.1	molecular chaperone Hsp33	1.42	0.07
**AFE_2773**	WP_012607524.1	thiol:disulfide oxidoreductase	1.42	0.00
**AFE_2665**	WP_009563301.1	molecular chaperone DnaK	1.34	0.00
**AFE_0553**	WP_012536200.1	thioredoxin family protein	1.33	0.22
**AFE_2372**	WP_012537156.1	type I restriction-modification system subunit M	1.21	0.01
**AFE_3116**	WP_012537283.1	Peroxiredoxin family protein	1.20	0.07
**AFE_0684**	WP_012536282.1	restriction endonuclease EcoEI subunit M	1.19	0.03
**AFE_3063**	WP_012537575.1	glutathione synthase	1.07	0.02
**AFE_2481**	WP_012607441.1	mercury(II) reductase	1.03	0.00
**AFE_2347**	WP_009563737.1	ferritin	0.93	0.10
**AFE_0677**	WP_012536278.1	molecular chaperone HscA	0.93	0.00
**AFE_3194**	WP_009566737.1	ATP-dependent DNA helicase RecG	0.91	0.00
**AFE_2526**	WP_012537242.1	N-carbamoylputrescine amidase	0.88	0.01
**AFE_1814**	WP_009563432.1	alkylhydroperoxidase	0.78	0.06
**AFE_3158**	WP_012537630.1	molecular chaperone HtpG	0.75	0.02
**AFE_1468**	WP_009562391.1	peroxiredoxin	0.75	0.00
**AFE_0657**	WP_012536267.1	thiol reductase thioredoxin	0.75	0.08
**AFE_0845**	WP_012536389.1	excinuclease ABC subunit B	0.72	0.14
**AFE_2738**	WP_009561099.1	Co2+/Mg2+ efflux protein ApaG	0.70	0.75
**AFE_0744**	WP_012536326.1	DNA mismatch repair protein MutS	0.67	0.07
**AFE_1760**	WP_012536740.1	DNA mismatch repair protein MutS	0.55	0.43
**AFE_0156**	WP_009567116.1	spermidine synthase	0.49	0.09
**AFE_0267**	WP_009565299.1	pyridine nucleotide-disulfide oxidoreductase	0.44	0.19
**AFE_2946**	WP_009560846.1	peptide-methionine (S)-S-oxide reductase	0.32	0.28
**AFE_1723**	WP_009563486.1	general stress protein	0.30	0.07
**AFE_1898**	WP_009566995.1	superoxide dismutase	0.17	0.54
**AFE_0004**	WP_012535741.1	pyridine nucleotide-disulfide oxidoreductase	0.17	0.41
**AFE_2848**	WP_012537429.1	thioredoxin	0.01	0.98

**AFE_0366**	WP_012536110.1	glutathione-disulfide reductase	-0.03	0.98
**AFE_0932**	WP_012536446.1	DNA recombination/repair protein RecA	-0.10	0.62
**AFE_0375**	WP_009566072.1	thioredoxin-disulfide reductase	-0.12	0.87
**AFE_0542**	WP_012536194.1	molecular chaperone GroEL	-0.12	0.58
**AFE_2362**	WP_012537150.1	thiol reductase thioredoxin	-0.15	0.78
**AFE_1872**	WP_012536812.1	thiol:disulfide interchange protein	-0.23	0.51
**AFE_2133**	WP_009566245.1	universal stress protein	-0.27	0.82
**AFE_2947**	WP_012607576.1	thiol:disulfide interchange protein	-0.29	0.25
**AFE_3038**	WP_012537561.1	monothiol glutaredoxin, Grx4 family	-0.36	0.51
**AFE_3118**	WP_009566821.1	pyridine nucleotide-disulfide oxidoreductase	-0.40	0.03
**AFE_0985**	WP_012536483.1	peroxidase	-0.50	0.03
**AFE_0006**	WP_012535742.1	chaperone protein EcpD	-0.62	0.08
**AFE_1682**	WP_012607171.1	bacterioferritin	-0.73	0.01
**AFE_2959**	WP_009564008.1	Fe(2+)-trafficking protein	-0.74	0.23
**AFE_0958**	WP_009565875.1	pyridine nucleotide-disulfide oxidoreductase	-0.76	0.03
**AFE_0543**	WP_009567579.1	molecular chaperone GroES	-0.83	0.12
**AFE_2847**	WP_012537428.1	excinuclease ABC subunit A	-0.86	0.28
**AFE_0199**	WP_012536015.1	ferritin	-1.03	0.04
**AFE_1548**	WP_012536596.1	globin	-1.50	0.05
**AFE_2246**	WP_012537078.1	disulfide isomerase	-1.69	0.05
**AFE_2664**	WP_012537323.1	molecular chaperone DnaJ	-1.76	0.01
**AFE_1943**	WP_012607270.1	disulfide isomerase	-1.77	0.02
**AFE_0367**	WP_009566864.1	glutathione amide-dependent peroxidase	-2.41	0.00
**AFE_1767**	WP_012536741.1	exodeoxyribonuclease X	-2.74	0.01
**AFE_3127**	WP_009567512.1	cobalamin biosynthesis protein CbiX	-5.38	0.00

Supplementary Table 3. Transcription affecting proteins and transcriptional regulators with enhanced expression in pyrite biofilm cells ($log_2 > 1$, q-value ≤ 0.10) and iron(II)-grown cells ($log_2 < -1$, q-value ≤ 0.10).

Locus tag	Protein ID	Description	log_2 pyrite/Fe	q-value
**AFE_2557**	WP_009562307.1	transcriptional regulator	3.06	0.00
**AFE_0135**	WP_012535969.1	LysR family transcriptional regulator	2.28	0.02
**AFE_0590**	WP_009564993.1	cold-shock protein	2.13	0.05
**AFE_2915**	WP_009567505.1	nitrogen regulatory protein P-II 1	2.02	0.00
**AFE_0429**	WP_009566205.1	nitrogen regulatory protein P-II	2.00	0.00
**AFE_0024**	WP_009566311.1	nitrogen assimilation regulatory protein NtrX	1.83	0.00
**AFE_3060**	WP_009566940.1	bifunctional pyr operon transcriptional regulator/uracil phosphoribosyltransferase	1.39	0.01
**AFE_0255**	WP_009566155.1	TetR family transcriptional regulator	1.12	0.01
**AFE_0314**	WP_012536079.1	transcription termination/antitermination protein NusG	-1.07	0.01
**AFE_0638**	WP_009564109.1	transcription elongation factor GreA	-1.14	0.01
**AFE_0061**	WP_009567692.1	Holliday junction branch migration protein RuvA	-1.16	0.09
**AFE_0215**	WP_012536023.1	division/cell wall cluster transcriptional repressor MraZ	-1.31	0.01
**AFE_0911**	WP_012536430.1	ATP-dependent Clp protease ATP-binding subunit ClpX	-1.83	0.00
**AFE_2336**	WP_012537129.1	RNA polymerase sigma factor RpoD	-1.85	0.01
**AFE_2158**	WP_009561657.1	LysR family transcriptional regulator	-2.30	0.01
**AFE_0391**	WP_012536117.1	translation initiation factor IF-2	-2.45	0.00
**AFE_1668**	WP_012607166.1	IclR family transcriptional regulator	-2.56	0.00
**AFE_1463**	WP_012536538.1	dihydroorotase	-3.10	0.00
**AFE_0055**	WP_009565541.1	FmdB family transcriptional regulator	-3.54	0.00
**AFE_1689**	WP_041645863.1	transcriptional initiation protein Tat	-3.83	0.00
**AFE_0059**	WP_009567690.1	transcriptional regulator	-4.15	0.00
**AFE_0353**	WP_012536098.1	30S ribosomal protein S4	-5.47	0.00

Supplementary Table 4. Identified proteins associated with carbon fixation in *A. ferrooxidans*[T]

gene	Locus tag	Protein ID	Description	log_2 pyrite/Fe	q-value
			**Putative operon cbb1**		
**cbbL1**	AFE_1691	WP_012536704.1	Ribulose bisphosphate carboxylase large chain 1	-2.27	0.00
**cbbS1**	AFE_1690	WP_009567528.1	ribulose bisphosphate carboxylase small subunit	-1.44	0.01
**csoS2**	AFE_1689	WP_041645863.1	transcriptional initiation protein Tat	-3.83	0.00
**csoS3**	AFE_1688	WP_012607174.1	carboxysome shell carbonic anhydrase	-1.61	0.01
**csoS4A**	AFE_1687	WP_009567526.1	carboxysome peptide A	1.03	0.71
**csoS4B**	AFE_1686	WP_009567525.1	carboxysome peptide B	0.97	0.08
**csoS1C**	AFE_1685	WP_012536700.1	carbon dioxide-concentrating protein CcmK	-0.25	0.31
**csoS1B**	AFE_1683	WP_012607172.1	carbon dioxide-concentrating protein CcmK	0.14	0.83
**bfrA**	AFE_1682	WP_012607171.1	bacterioferritin	-0.73	0.01
**hyp1**	AFE_1681	WP_012536697.1	hypothetical protein	-1.40	0.01
**parA**	AFE_1680	WP_012607170.1	cobyrinic acid a,c-diamide synthase	-1.09	0.01
**hyp2**	AFE_1679	ACK78664.1	hypothetical protein	-3.81	0.01
**cbbQ1**	AFE_1678	WP_012536694.1	ATPase AAA	0.17	0.59
**cbbO1**	AFE_1677	WP_012536693.1	VWA domain-containing protein	0.00	0.98
**cbbA**	AFE_1676	WP_009561318.1	fructose-bisphosphate aldolase	-1.84	0.00
			**Putative operon cbb2**		
**cbbL2**	AFE_3051	WP_009566926.1	Ribulose bisphosphate carboxylase large chain 2	2.43	0.00
**cbbS2**	AFE_3052	WP_009566929.1	ribulose bisphosphate carboxylase small subunit	2.55	0.00
**cbbQ2**	AFE_3053	WP_009566930.1	ATPase AAA	1.51	0.00

**cbbO2**	AFE_3054	WP_009566932.1	VWA domain-containing protein	1.23	0.10
			**Putative operon cbb3**		
**cbbF**	AFE_3253	WP_012537679.1	fructose 1,6-bisphosphatase	-0.88	0.02
**cbbT**	AFE_3252	WP_012537678.1	transketolase	0.27	0.09
**cbbG**	AFE_3251	WP_009567621.1	type I glyceraldehyde-3-phosphate dehydrogenase	-0.21	0.17
**cbbK**	AFE_3250	WP_012537677.1	phosphoglycerate kinase	-0.18	0.28
**pykA**	AFE_3249	WP_012537676.1	pyruvate kinase	-1.42	0.00
**cbbA**	AFE_3248	WP_009561009.1	fructose-1,6-bisphosphate aldolase	-0.05	0.81
**cbbE**	AFE_3247	WP_012537675.1	ribulose-phosphate 3-epimerase	0.98	0.05
**cbbZ**	AFE_3246	WP_009561007.1	phosphoglycolate phosphatase, bacterial	0.31	0.66
**trpE**	AFE_3245	WP_012537674.1	anthranilate synthase component I	1.06	0.13
**trpG**	AFE_3244	WP_009567142.1	glutamine amidotransferase	-0.12	0.87
			**Putative operon cbb4**		
**metK**	AFE_0532	WP_012536187.1	S-adenosylmethionine synthase	0.71	0.02
**sahA**	AFE_0534	WP_009567191.1	adenosylhomocysteinase	-0.51	0.02
**metF**	AFE_0535	WP_012536188.1	methylenetetrahydrofolate reductase [NAD(P)H]	1.57	0.01
**cbbP**	AFE_0536	WP_012536189.1	phosphoribulokinase	-0.97	0.03
			**Putative operon cbb5**		
**cbbM**	AFE_2155	WP_012537012.1	ribulose-bisphosphate carboxylase	-0.04	0.94
**cbbQ3**	AFE_2156	WP_012537013.1	ATPase AAA	-0.88	0.08
**$cbbR_M$**	AFE_2158	WP_009561657.1	LysR family transcriptional regulator	-2.30	0.01

Supplementary Table 5. Proteins in COGs cell wall/membrane biogenesis (M), carbohydrate transport and metabolism (G), intracellular trafficking and secretion (U), lipid transport and metabolism (I) and cell motility (N) with enhanced expression in pyrite biofilm cells ($log_2 > 1$, q-value ≤ 0.10) and iron(II)-grown cells ($log_2 < -1$, q-value ≤ 0.10).

Locus tag	Protein ID	Description	log_2 pyrite/Fe	q-value
		**Cell wall/membrane biogenesis M**		
**AFE_2967**	WP_012607583.1	group 1 glycosyl transferase	3.04	0.04
**AFE_2316**	WP_012537117.1	membrane protein	3.01	0.10
**AFE_3264**	WP_012607659.1	RND transporter	1.96	0.00
**AFE_1552**	WP_012536600.1	sucrose synthase	1.89	0.01
**AFE_3291**	WP_012537698.1	NAD-dependent dehydratase	1.73	0.00
**AFE_3265**	WP_012537684.1	transporter	1.46	0.02
**AFE_1880**	WP_009564623.1	RND transporter	1.44	0.00
**AFE_2368**	WP_012537154.1	transporter	1.27	0.01
**AFE_2923**	WP_012607567.1	peptidase M15	1.20	0.00
**AFE_2367**	WP_012537153.1	RND transporter	1.16	0.03
**AFE_2677**	WP_009565767.1	3-deoxy-D-manno-octulosonic acid transferase	1.14	0.01
**AFE_0442**	WP_012536140.1	NAD(P)-dependent oxidoreductase	1.12	0.03
**AFE_2358**	WP_009561191.1	4-hydroxy-tetrahydrodipicolinate synthase	1.10	0.01
**AFE_0848**	WP_012536391.1	2-dehydro-3-deoxyphosphooctonate aldolase	1.07	0.04
**AFE_1879**	WP_012536818.1	MexH family multidrug efflux RND transporter periplasmic adaptor subunit	0.97	0.01
**AFE_2113**	WP_012536981.1	VacJ family lipoprotein	-0.92	0.01
**AFE_2186**	WP_009561052.1	prolipoprotein diacylglyceryl transferase	-1.04	0.02
**AFE_2961**	WP_012537503.1	capsule polysaccharide export inner-membrane protein	-1.09	0.01
**AFE_0524**	WP_012536181.1	peptidase S41	-1.18	0.01
**AFE_3034**	WP_009562824.1	organic solvent tolerance protein	-1.70	0.10
**AFE_0377**	WP_009566070.1	outer membrane lipoprotein carrier protein LolA	-1.71	0.00
**AFE_3293**	WP_012537700.1	mannose-1-phosphate	-1.74	0.01

		guanylyltransferase/mannose-6-phosphate isomerase		
**AFE_2929**	WP_012537480.1	cyclopropane-fatty-acyl-phospholipid synthase	-1.89	0.00
**AFE_2883**	WP_012537451.1	choloylglycine hydrolase	-2.23	0.10
**AFE_2223**	WP_012537062.1	penicillin-binding protein 1A	-2.74	0.01
**AFE_1397**	WP_009567236.1	elongation factor 4	-2.96	0.01
**AFE_2268**	WP_012537091.1	cell envelope biogenesis protein TonB	-3.32	0.00
**AFE_1456**	WP_012536532.1	acyl-[acyl-carrier-protein]--UDP-N-acetylglucosamine O-acyltransferase	-3.59	0.01
**AFE_2267**	WP_012607367.1	phospholipase C	-4.31	0.00
**AFE_1827**	WP_041646068.1	murein transglycosylase	-5.74	0.00
		**Carbohydrate transport and metabolism, G**		
**AFE_1799**	WP_012536767.1	alpha-glucan phosphorylase	3.95	0.01
**AFE_1802**	WP_012536769.1	aldolase	3.66	0.03
**AFE_1843**	WP_012536797.1	transketolase	3.41	0.00
**AFE_1815**	WP_012536777.1	2,3-bisphosphoglycerate-independent phosphoglycerate mutase	2.74	0.00
**AFE_0451**	WP_012606559.1	NAD kinase	2.67	0.02
**AFE_3051**	WP_009566926.1	Ribulose bisphosphate carboxylase large chain 2	2.43	0.00
**AFE_2836**	WP_009562056.1	glycogen-branching enzyme	2.32	0.01
**AFE_3302**	WP_009566480.1	HIT family protein	2.07	0.00
**AFE_3018**	WP_012537546.1	phosphoenolpyruvate--protein phosphotransferase	1.84	0.00
**AFE_0419**	WP_012536126.1	transaldolase	1.74	0.00
**AFE_1816**	WP_012536778.1	phosphoenolpyruvate synthase	1.69	0.00
**AFE_0669**	WP_009564866.1	inositol monophosphatase	1.67	0.10
**AFE_3023**	WP_012537551.1	PTS sugar transporter	1.56	0.01
**AFE_2324**	WP_012537123.1	alpha-D-glucose phosphate-specific phosphoglucomutase	1.53	0.00
**AFE_2025**	WP_012536916.1	glucose-6-phosphate dehydrogenase	1.34	0.01
**AFE_2839**	WP_012537423.1	glycoside hydrolase	1.24	0.00
**AFE_0074**	WP_012606487.1	chain-length determining protein	1.23	0.01

**AFE_1923**	WP_012536840.1	NAD(P)H-hydrate dehydratase	1.14	0.09
**AFE_2009**	ACK78253.1	sugar ABC transporter [WP_072452544.1, E-value: $2 \cdot 10^{-50}$]	1.12	0.04
**AFE_2678**	WP_009565766.1	starch synthase	1.07	0.00
**AFE_3020**	WP_012537549.1	PTS sugar transporter subunit IIA	1.03	0.01
**AFE_2224**	WP_012537063.1	glycosyl hydrolase	1.00	0.01
**AFE_0629**	WP_009560936.1	ribose-5-phosphate isomerase	0.99	0.02
**AFE_3247**	WP_012537675.1	ribulose-phosphate 3-epimerase	0.98	0.05
**AFE_0434**	WP_012536136.1	2,3-diketo-5-methylthiopentyl-1-phosphate enolase	0.95	0.06
**AFE_0138**	WP_012535972.1	lipopolysaccharide heptosyltransferase II	-0.96	0.01
**AFE_2634**	WP_009566647.1	phosphoglucosamine mutase	-1.11	0.02
**AFE_3249**	WP_012537676.1	pyruvate kinase	-1.42	0.00
**AFE_1766**	WP_009566988.1	aldolase	-1.44	0.03
**AFE_0082**	WP_012535780.1	rhamnosyltransferase	-1.62	0.01
**AFE_1676**	WP_009561318.1	fructose-bisphosphate aldolase	-1.84	0.00
**AFE_1667**	WP_009565308.1	transketolase	-1.98	0.00
**AFE_1691**	WP_012536704.1	Ribulose bisphosphate carboxylase large chain 1	-2.27	0.00
**AFE_0081**	WP_012535779.1	mannosyltransferase	-3.09	0.01
		**Intracellular trafficking and secretion, U**		
**AFE_1915**	WP_009561366.1	pilus assembly protein PilZ	2.23	0.07
**AFE_0286**	WP_012536065.1	sulfate permease membrane protein	1.93	0.02
**AFE_3265**	WP_012537684.1	outer membrane efflux protein	1.46	0.02
**AFE_1880**	WP_009564623.1	RND efflux system, outer membrane lipoprotein, NodT family	1.44	0.00
**AFE_2368**	WP_012537154.1	outer membrane efflux protein	1.27	0.01
**AFE_2753**	WP_009568264.1	signal recognition particle-docking protein FtsY	1.12	0.03
**AFE_0910**	WP_012536429.1	ATP-dependent Clp protease proteolytic subunit	0.98	0.02
**AFE_2057**	WP_012536937.1	protein-export membrane protein SecD	0.94	0.01
**AFE_1398**	WP_009567235.1	S26 family signal peptidase	-1.01	0.07
**AFE_0736**	WP_012536320.1	pilus assembly protein PilP	-1.08	0.03

**AFE_3049**	WP_009566923.1	twin arginine-targeting protein translocase TatB	-1.18	0.04
**AFE_0737**	WP_012536321.1	pilus assembly protein PilO	-1.35	0.00
		**Lipid transport and metabolism, I**		
**AFE_1857**	WP_012536805.1	glucose dehydrogenase	4.75	0.00
**AFE_1651**	WP_012536669.1	3-hydroxyisobutyrate dehydrogenase family protein	3.19	0.00
**AFE_2542**	WP_012537248.1	phosphatidylserine decarboxylase	3.10	0.00
**AFE_0748**	WP_009562365.1	glucose 1-dehydrogenase, putative	2.02	0.01
**AFE_2225**	WP_012537064.1	acetyl-CoA synthetase	1.36	0.01
**AFE_1908**	WP_009561353.1	acetyl-CoA carboxylase, carboxyl transferase, beta subunit	-0.61	0.03
**AFE_3258**	WP_009566635.1	holo-(acyl-carrier-protein) synthase	-0.68	0.06
**AFE_1404**	WP_009565421.1	acetyl-CoA carboxylase, carboxyl transferase, alpha subunit	-0.92	0.04
**AFE_1462**	WP_012536536.1	enoyl-(acyl-carrier-protein) reductase	-1.05	0.10
**AFE_0420**	WP_009562995.1	SCP-2 sterol transfer family protein	-1.14	0.00
**AFE_1905**	WP_009566798.1	fatty acid/phospholipid synthesis protein PlsX	-1.84	0.01
**AFE_1448**	WP_012536524.1	undecaprenyl diphosphate synthase	-2.03	0.01
**AFE_1933**	WP_009564018.1	4-hydroxy-3-methylbut-2-en-1-yl diphosphate synthase (flavodoxin)	-2.31	0.00
**AFE_3108**	WP_009566406.1	short-chain dehydrogenase	-2.51	0.00
		**Cell motility, N**		
**AFE_1915**	WP_009561366.1	pilus assembly protein PilZ	2.23	0.07
**AFE_0736**	WP_012536320.1	pilus assembly protein PilP	-1.08	0.03
**AFE_0737**	WP_012536321.1	pilus assembly protein PilO	-1.35	0.00
**AFE_0265**	WP_012536053.1	twitching motility protein PilT	-1.19	0.01
